# WHIRLIES Are Multifunctional DNA-Binding Proteins With Impact on Plant Development and Stress Resistance

**DOI:** 10.3389/fpls.2022.880423

**Published:** 2022-04-21

**Authors:** Karin Krupinska, Christine Desel, Susann Frank, Götz Hensel

**Affiliations:** ^1^Institute of Botany, Christian-Albrechts-University of Kiel, Kiel, Germany; ^2^Centre for Plant Genome Engineering, Institute of Plant Biochemistry, Heinrich-Heine-University Düsseldorf, Düsseldorf, Germany; ^3^Centre of Region Haná for Biotechnological and Agricultural Research, Czech Advanced Technology and Research Institute, Palacký University Olomouc, Olomouc, Czechia

**Keywords:** DNA-binding, nucleoid, stress, development, WHIRLY

## Abstract

WHIRLIES are plant-specific proteins binding to DNA in plastids, mitochondria, and nucleus. They have been identified as significant components of nucleoids in the organelles where they regulate the structure of the nucleoids and diverse DNA-associated processes. WHIRLIES also fulfil roles in the nucleus by interacting with telomers and various transcription factors, among them members of the WRKY family. While most plants have two WHIRLY proteins, additional WHIRLY proteins evolved by gene duplication in some dicot families. All WHIRLY proteins share a conserved WHIRLY domain responsible for ssDNA binding. Structural analyses revealed that WHIRLY proteins form tetramers and higher-order complexes upon binding to DNA. An outstanding feature is the parallel localization of WHIRLY proteins in two or three cell compartments. Because they translocate from organelles to the nucleus, WHIRLY proteins are excellent candidates for transducing signals between organelles and nucleus to allow for coordinated activities of the different genomes. Developmental cues and environmental factors control the expression of WHIRLY genes. Mutants and plants with a reduced abundance of WHIRLY proteins gave insight into their multiple functionalities. In chloroplasts, a reduction of the WHIRLY level leads to changes in replication, transcription, RNA processing, and DNA repair. Furthermore, chloroplast development, ribosome formation, and photosynthesis are impaired in monocots. In mitochondria, a low level of WHIRLIES coincides with a reduced number of cristae and a low rate of respiration. The WHIRLY proteins are involved in the plants’ resistance toward abiotic and biotic stress. Plants with low levels of WHIRLIES show reduced responsiveness toward diverse environmental factors, such as light and drought. Consequently, because such plants are impaired in acclimation, they accumulate reactive oxygen species under stress conditions. In contrast, several plant species overexpressing WHIRLIES were shown to have a higher resistance toward stress and pathogen attacks. By their multiple interactions with organelle proteins and nuclear transcription factors maybe a comma can be inserted here? and their participation in organelle–nucleus communication, WHIRLY proteins are proposed to serve plant development and stress resistance by coordinating processes at different levels. It is proposed that the multifunctionality of WHIRLY proteins is linked to the plasticity of land plants that develop and function in a continuously changing environment.

## Introduction

Plant-specific WHIRLY proteins received increasing attention in recent years regarding their participation in development and stress resistance. WHIRLY proteins are DNA/RNA-binding proteins sharing a highly conserved WHIRLY DNA-binding domain and are present in all DNA containing compartments of the plant cell. Higher plants share a KGKAAL motif in the WHIRLY domain that mediates the binding to single-stranded DNA (ssDNA). WHIRLY-like proteins with a high structural similarity but lacking the KGKAAL motif are also found in green algae, such as *Ostreococcus taurii* (Krause et al., 2009), *Klebsormidium flaccidum*, a member of the Charophyceae that are the closest relatives of land plants, and in the liverwort *Marchantia polymorpha*, a representative of the most basal plant lineage (Kobayashi et al., 2016). While the algae and Marchantia have only one WHIRLY-like protein, higher plant species have at least two WHIRLY proteins. Both have an organelle targeting peptide at the N-terminus, directing them either to mitochondria, plastids, or both organelles (Krause et al., 2005; Golin et al., 2020).

WHIRLY1 was initially identified as the 24 kDa protein (p24) subunit of a factor binding to the promoter of the *PR-10a* gene of potato and hence named *PR-10a* binding factor 2 (PBF-2; Desveaux et al., 2000). By crystallographic analyses, PBF-2 was shown to be a p24 tetramer having a structure compared with whirligigs (Desveaux et al., 2002). A structural comparison of WHIRLY proteins from potato and Arabidopsis revealed that they share a conserved orientation of different residues providing a platform for binding ssDNA in a non-sequence-specific manner (Cappadocia et al., 2013).

The literature on WHIRLY proteins is relatively diverse, reporting on seemingly unrelated features of the members of the WHIRLY family. To gain a comprehensive understanding of the biological significance of WHIRLIES, this review aims at integrating the different findings on these multifunctional proteins.

## Conservation and Variation of Whirly Sequences

Whereas most higher plants have two WHIRLY proteins, *Arabidopsis thaliana* and other members of the Brassicaceae family have three WHIRLIES, of which WHIRLY1 is targeted to chloroplasts, WHIRLY2 to mitochondria, and WHIRLY3 is dually targeted to both organelles (Krause et al., 2005; Golin et al., 2020). In different strawberry genomes, gene duplication has resulted in the presence of up to five *WHIRLY* genes (Hu and Shu, 2021).

**Figure 1:**
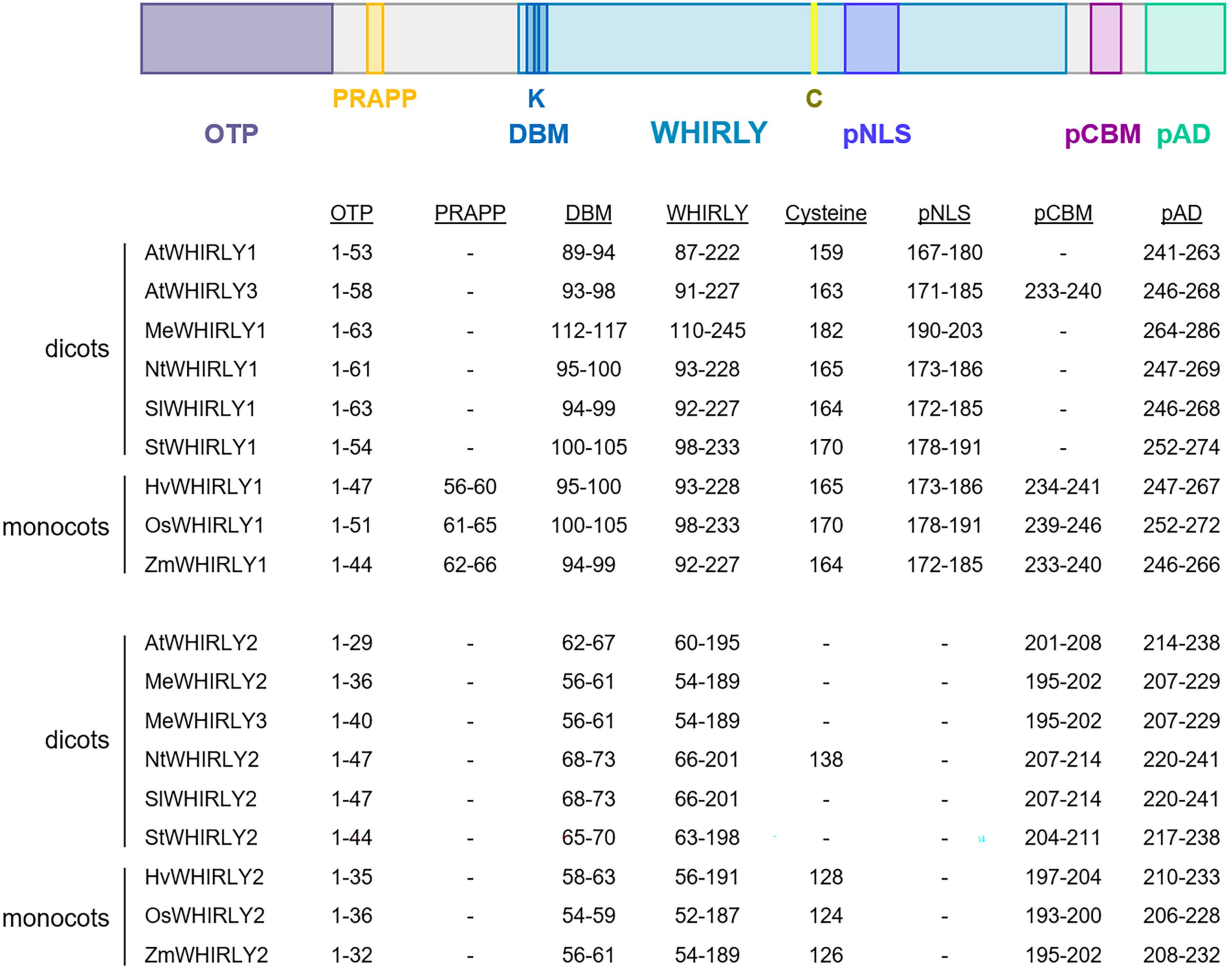
Schematic comparison of motifs in selected WHIRLY proteins. AtWHIRLY1 (*Arabidopsis thaliana*, NP_172893), AtWHIRLY2 (*A. thaliana*, NP_177282.2), AtWHIRLY3 (*A. thaliana*, NP_178377), HvWHIRLY1 (*Hordeum vulgare*, BAJ96655), HvWHIRLY2 (*H. vulgare*, BF627441.2), SlWHIRLY1 (*Solanum lycopersicum*, AFY24240.1), SlWHIRLY2 (*S. lycopersicum*, XP_010313085.1), StWHIRLY1 (*Solanum tuberosum*, NP_001275155.1), StWHIRLY2 (*S. tuberosum*, NP_001275393.1), NtWHIRLY1 (*Nicotiana tabacum*, XP_016453689.1), NtWHIRLY2 (*N. tabacum*, XP_016511175.1), ZmWHIRLY1 (*Zea mays*, NP_001123589.1), ZmWHY2 (*Z. mays*, NP_001152589.2), OsWHIRLY1 (*Oryza sativa*, BAD68418.1), OsWHIRLY2 (*O. sativa*, NP_001045956.1). The WHIRLY domain is colored in light blue. The N-terminal organelle targeting peptides (OTP) are illustrated in grey. Below the scheme, the positions of selected amino acid residues and motifs in different WHIRLIES are listed: the PRAPP motif in yellow, the DNA-binding motif (DBM) and a putative nuclear localization motif (pNLS) in blue, a putative copper-binding motif in purple and a putative transactivation domain (pAD) in green. The organelle targeting peptides (OTP) were predicted with TargetP-2.0 (https://services.healthtech.dtu.dk/service.php?TargetP-2.0) or UniProt, (https://www.uniprot.org/), the putative nuclear localization signal (pNLS) with NLStradamus (http://www.moseslab.csb.utoronto.ca/NLStradamus/) and the putative copper-binding motif (CBM) with Motif Scan (https://myhits.sib.swiss/cgi-bin/motif_scan). The putative autoregulatory domain (pAD) was defined by Desveaux et al. (2005).

The KGKAAL motif shared by all WHIRLY proteins in higher plants was shown to be required for binding to ssDNA and for the hexamerization of the tetramers resulting in hollow sphere structures of 12 nm in diameter (Cappadocia et al., 2012). These sphere-like structures were shown to further assemble into large protein DNA complexes by DNA-dependent joining of adjacent spheres (Cappadocia et al., 2012). Besides the DNA-binding motif (DBM), that is, KGKAAL (or KGKAAM in the case of the rice WHIRLY1), many WHIRLY proteins share a conserved cysteine whose function may be essential for redox regulation (Foyer et al., 2014). Additionally, a putative nuclear localization sequence can be found in the WHIRLY domain of plastid-targeted WHIRLY proteins (Figure 1). However, the functionality of this motif is questionable considering that AtWHIRLY:GFP fusion proteins are not imported into the nucleus (Krause et al., 2005).

Sequence comparisons of WHIRLY proteins from different species revealed that WHIRLY proteins possess additional motifs in the variable sequences preceding the WHIRLY domain (Desveaux et al., 2005). This result suggests that the proteins might have different functions in different species. N-terminal polyglutamine stretches shared by several dicot WHIRLY1 proteins (*St*, *Le*, *Vv*), the alternative proline-rich sequences of monocot WHIRLY1 proteins, and the serine-rich sequences of members of the Brassicaceae family were predicted to serve as transactivation motifs (Desveaux et al., 2005). The mutation of different WHIRLY sequences revealed, however, that the proline-rich PRAPP motif of monocot WHIRLY1 proteins rather contributes to the compaction of organellar nucleoids (Oetke et al., 2022).

In the C-terminal variable parts of the WHIRLY sequences, putative autoregulatory domains (pAD) have been identified (Desveaux et al., 2005). In the crystal structure of StWHIRLY1, it is evident that Glu271 and Trp272 in the C-terminal part interact with Lys188 of the WHIRLY domain. Desveaux and Brisson observed that a mutation of Trp272 resulted in a higher ssDNA-binding activity *in vitro*, suggesting that the C-terminal region might interfere with the binding to ssDNA (Desveaux et al., 2005). Sequence alignments show that Lys188 and the C-terminal Glu271 and Trp272 of StWHIRLY1 are highly conserved in all WHIRLY proteins (Supplementary Figure 1). Furthermore, many WHIRLY proteins possess a putative copper-binding motif (pCBM) preceding a putative transactivation domain (pAD; Figure 1), which has also been found in the prion protein, where it is crucial for the protection of neurons (Prince and Gunson, 1998; Nguyen et al., 2019). The pCBM is a motif shared by all monocot WHIRLIES while it is lacking in the dicot WHIRLY1 sequences. In Arabidopsis, WHIRLY2 and WHIRLY3 share this motif.

The WHIRLY sequences contain several putative sites for posttranslational modifications, including phosphorylation and sumoylation (Grabowski et al., 2008). Matching these predictions, several studies report on different molecular weights of WHIRLY proteins. For example, WHIRLY1 has been detected in nuclei prepared from Arabidopsis leaves with a higher molecular weight of 29 kDa instead of 24 kDa (Lin et al., 2020). In tomato nuclei, a WHIRLY1 with the predicted size of the plastid form coexists with an additional form of higher molecular weight (Zhuang et al., 2019). The conditions leading to these putative modifications of WHIRLY1 remain to be determined. In potato, the p24 subunit of PBF, that is, WHIRLY1, was reported to be phosphorylated when binding to the *PR-10a* promoter in the nucleus (Després et al., 1995). Phosphorylation by a homolog of mammalian protein kinase C was essential for transcriptional activation of the *PR-10a* gene by PBF-2 (Subramaniam et al., 1997; Desveaux et al., 2000). Recombinant Arabidopsis WHIRLY2 was shown to be phosphorylated by a mitochondrial kinase (Tarasenko et al., 2012).

## Subcellular Targeting and Localization of Whirly Proteins

For Arabidopsis, it has been shown that WHIRLY1 in fusion with the green fluorescence protein (GFP) is targeted to chloroplasts while WHIRLY2:GFP is targeted to mitochondria (Krause et al., 2005). *In organello*, import assays showed that AtWHIRLY2 is also imported into chloroplasts (Krause et al., 2005). The third WHIRLY protein of *A. thaliana* is imported into both chloroplasts and mitochondria (Golin et al., 2020). When the WHIRLY genes in fusion with fluorescent proteins were stably overexpressed in Arabidopsis, both WHIRLY1 and WHIRLY3 were exclusively detected in chloroplasts and WHIRLY2 in mitochondria, respectively (Isemer, 2013). The discrepancies observed between the localization of the fusion proteins and the *in organello* import assays might be due to changes in size and conformation resulting from the fusion with the fluorescent protein (Sharma et al., 2018). Neither protoplasts nor transgenic plants were able to show the nuclear localization of any Arabidopsis WHIRLY protein in fusion with a fluorescent protein.

**Figure 2:**
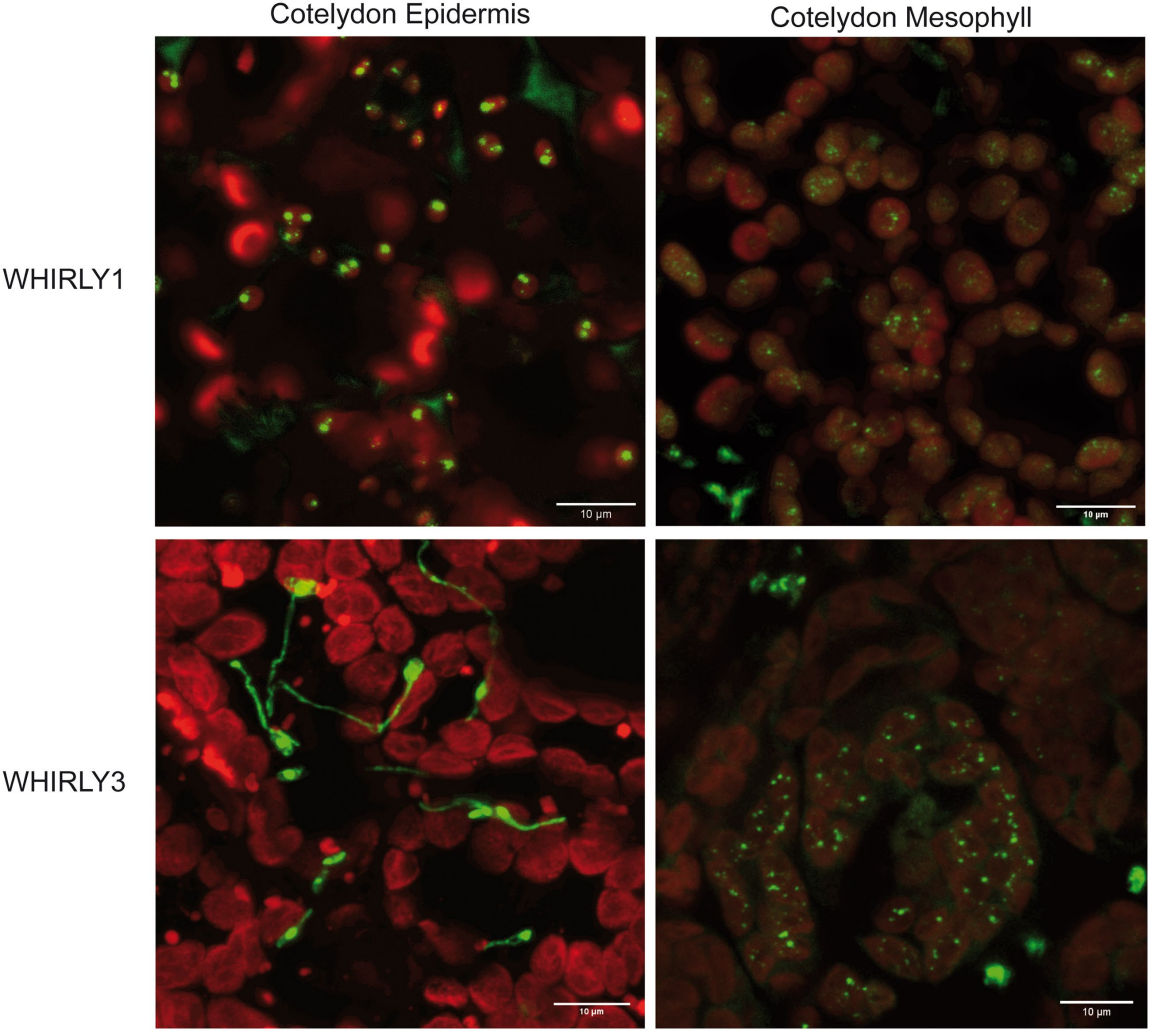
Detection of WHIRLY1:GFP and WHIRLY3:RFP fusion proteins in epidermal cells of the cotyledons of stably transformed Arabidopsis plants. The constructs were overexpressed under the control of the CaMV *35S* promoter. The constructs were overexpressed under the control of the CaMV *35S* promoter. Leaves were imbedded in PBS:glycerol (1:1) without fixation and analyzed by a LEICA SP5 laser scanning microscope and a HCX PL Apo 63x/1.2 W objective. Sequential scans per frame for GFP [Ex 488 nm (6%), Em 510–550 nm], mRFP [Ex 543nm (14%), Em 580–610nm] and chlorophyll [Ex 633 (5%), Em 690–750 nm] were carried out. Chlorophyll fluorescence is shown in red while signals of WHIRLY1:GFP and WHIRLY3:RFP displayed both in green. A projection out of five optical layers representing 3 μm in z-direction are created by the LAS X software. The bars represent 10 μm each.

Although WHIRLY1:GFP and WHIRLY3:RFP fusion proteins were both detected in chloroplasts, they showed differences concerning their distribution in the plastids of different tissues (Figure 2). While in mesophyll cells, WHIRLY1 and 3 showed both the typical nucleoid association, in epidermal cells, WHIRLY3:RFP fluorescence was also seen in the frequently formed stromules (Figure 2). This finding indicates that the gene duplication in Arabidopsis gave rise to a specialization of the different plastid-targeted WHIRLY proteins.

The nuclear localization of WHIRLY1 has so far only been deduced from immunological analysis and Edman degradation protein sequencing of the purified PBF-2 fraction from potato tubers (Desveaux et al., 2000), and from mass spectrometry of proteins binding to single-stranded telomere sequences (Yoo et al., 2007) and of the components of the KPRE binding factor 1 binding to the kinesin gene of Arabidopsis (Xiong et al., 2009). Immunogold analysis with a specific antibody directed toward WHIRLY1 in barley showed that the WHIRLY1 protein is dually located in the same cell’s chloroplasts and nucleus (Grabowski et al., 2008). Intriguingly, both isoforms have the same molecular weight as shown by immunoblot analysis of fractions from barley and maize leaves (Grabowski et al., 2008; Zhang et al., 2013), indicating that nuclear WHIRLY1 must first be processed inside chloroplasts. Indeed, in transplastomic tobacco plants synthesizing an HA-tagged form of the Arabidopsis WHIRLY1 protein in chloroplasts, WHIRLY1 was shown to translocate to the nucleus (Isemer et al., 2012b), where it acts as an activator of pathogen response genes as shown before for the potato WHIRLY1, that is, p24 (Desveaux et al., 2000). WHIRLY1 thereby is one of the few plant echoproteins described so far. These are proteins that have the same molecular weight in different compartments (Yogev and Pines, 2011; Krupinska et al., 2020). In Arabidopsis, WHIRLY2 was reported to have a triple localization in chloroplasts, mitochondria, and nucleus, with the nuclear form having a higher molecular weight (Huang et al., 2020). Putative posttranslational modifications associated with multiple compartmentation of the WHIRLY proteins remain to be identified.

## Specificity of the Binding of Whirlies to DNA

In the nucleus, WHIRLY1 has been found to bind to promoters of genes associated with stress responses and senescence (Table 1). In the first report on WHIRLY1, the 24 kDa protein had been identified as a subunit of the transcriptional activator complex PBF-2 binding to the potato *PR-10a* gene promoter (Desveaux et al., 2000). A 30-bp sequence in the promoter containing an inverted repeat sequence required for binding WHIRLY1 to the *PR10-a* promoter, that is, TGACANNNNTGTCA, has been named elicitor response element (ERE). Later on, mutational analyses have shown that the GTCAAAAA/T sequence is sufficient for ERE activity (Desveaux et al., 2004). This so-called PB (PBF-2 binding) element has been detected in the promoters of several defense genes and can overlap with the W-box (T/G)TGAC(C/T) and the TGACG element that are targets of WRKY and TGA family transcription factors, respectively (Desveaux et al., 2005). Furthermore, WHIRLY1 was identified as a repressor of senescence-associated genes, that is, *S40* of barley and *WRKY53* of *A. thaliana* (Miao et al., 2013; Krupinska et al., 2014a). The promoter of *HvS40* contains two ERE-like elements, one of them overlapping with two W-boxes (Krupinska et al., 2014a). In the promoter of *AtWRKY53*, WHIRLY1 was shown to bind to GNNNAAATT plus an AT-rich telomeric repeat-like sequence (Miao et al., 2013). Recent research with tomato plants revealed that WHIRLY1 binds to ERE-like sequences in promoters of genes involved in starch metabolism (Zhuang et al., 2019). By opposite effects on the expression of genes encoding starch degrading and starch synthesizing enzymes, WHIRLY1 contributes to an increase in soluble sugar content coinciding with enhanced chilling resistance (Zhuang et al., 2019). In Arabidopsis, WHIRLY1 was found to attach to telomers consisting of repeated TTTAGGG sequences (Yoo et al., 2007). Together with WHIRLY3, it was detected in a nuclear fraction binding to the promoter of the *KINESIN* gene, thereby repressing transcription (Xiong et al., 2009). The TGAGG(G/A) element in the *KINESIN* promoter (Table 1) overlaps with the W-box (T/G)TGAC(C/T) and the TGACG element recognized by the TGA family of transcription factors (Xiong et al., 2009). Overlapping of binding motifs used by WHIRLY1 and other transcription factors is in accordance with the identification of TGA1 and WRKY factors as interacting proteins of WHIRLIES (Supplementary Table 1).

**Table 1:** Target sequences of WHIRLY proteins in different plant species.

WHIRLY	Species	Target gene	Sequence	Method	References
WHIRLY1	*Solanum tuberosum*	*PR10-a*	ERE: TGACANNNNTGTCA	EMSA	Desveaux et al., 2000
WHIRLY1	*Solanum tuberosum*	*PR10-a*	PB element GTCAAAAA/T	ChIP	Desveaux et al., 2004
WHIRLY1	*Arabidopsis thaliana*	*Telomer*	TTTAGGG	EMSA	Yoo et al., 2007
WHIRLY1 WHIRLY3	*Arabidopsis thaliana*	*KINESIN KP1*	Promoter element KPRE including TGAGG(G/A)	ChIP	Xiong et al., 2009
WHIRLY1	*Arabidopsis thaliana*	*WRKY53*	GNNNAAATT plus an AT-rich telomeric repeat-like sequence	ChIP	Huang et al., 2018a
WHIRLY1	*Hordeum vulgare*	*HvS40*	ERE-like sequences and **W-boxes:** **TGTCA**GAAAT**GGTCAA** **GTCAA**ATAAT**GGTCAA**	EMSA	Krupinska et al., 2014a
WHIRLY1	*Lycopersicum esculentum*	*SlISA2*, *s*tarch synthesis-related gene	ERE	ChIP	Zhuang et al., 2019
WHIRLY1	*Lycopersicum esculentum*	*SlAMY3*, α-amylase	ERE-like	ChIP	Zhuang et al., 2019
WHIRLY1	*Lycopersicum esculentum*	*HSP21.5A*	ERE-like TGACACGTGGCAAT	ChIP Yeast 1HLUC expression	Zhuang et al., 2020a
WHIRLY1	*Lycopersicum esculentum*	*psbA*	GTTACCCT	Yeast 1H	Zhuang et al., 2019

Beyond this, the DNA sequences WHIRLIES bind to, share only little similarity (Table 1). Several binding motifs overlap with sequences used by other defense-related transcription factors. This might indicate an interplay between the ssDNA-binding WHIRLY and the transcription factors binding to double-stranded DNA (Desveaux et al., 2005; Krupinska et al., 2014a). It is known that several ssDNA-binding proteins regulate gene expression both positively and negatively (Rothman-Denes et al., 1998). Desveaux et al. (2005) proposed that ssDNA-binding proteins might sense twisted DNA produced during transcription and amplify gene transcription by their transactivation domains. Electrophoretic mobility shift assays (EMSA) with the ERE of *StPR-10a* and the ERE-like elements of the *HvS40* promoter revealed that the binding of WHIRLY1 is most robust to ssDNA and that the binding strength differed between coding and non-coding strands (Desveaux et al., 2000; Krupinska et al., 2014a). The *S40* promoter of barley contains two ERE-like elements in the promotor, which were shown to bind WHIRLY1 by electrophoretic mobility shift assays. While in senescent leaves a complex was formed only with the first motif, in non-senescent leaves a complex was formed with the second motif (Krupinska et al., 2014a). The results indicate that at least WHIRLY1 can function as repressor as well as activator of nuclear genes. Its activity might depend on interactions with other transcription factors, such as the WRKY factors binding to W-boxes included in the EREs (Ciolkowski et al., 2008).

In the organelles, the binding of WHIRLIES to DNA is somewhat independent of the DNA sequence as shown by immunoprecipitation with plastid DNA (Prikryl et al., 2008) and mitochondrial DNA (Maréchal et al., 2008; Tarasenko et al., 2012). An exception reported for tomato is its binding to the GTTACCCT sequence in the *psbA* promoter, which is proposed to ensure high expression of the *psbA* gene during chilling (Zhuang et al., 2019).

## Whirlies Control Chloroplast Development, Senescence, Embryo Development, and Germination

Arabidopsis T-DNA insertion mutants *why1* and *why2* as well as the tilling mutant of *WHIRLY3 (tilwhy3)*, do not show apparent changes in development (Yoo et al., 2007; Maréchal et al., 2008, 2009). Only a few plants of the progeny of the *why1tilwhy3* double mutant show variegated leaves indicating a disturbance of chloroplast development (Maréchal et al., 2009). Although several dicot species have been employed for silencing or overexpression of *WHIRLY* genes (Table 2), none of these studies reported a disturbance of chloroplast development.

**Table 2:** Phenotypes of plants with altered expression of WHIRLY genes, oe, overexpression, RNAi, RNA inference.

Species	Genetic modification	Development	Stress resistance	References
*Arabidopsis thaliana*	*why1* T-DNA mutant	Accelerated senescence		Miao et al., 2013
*Arabidopsis thaliana*	*why1 tilwhy3*	Leaf variegation		Maréchal et al., 2009
*Arabidopsis thaliana*	*tilwhy1* mutants		Reduced resistance to *Peronospora parasitica*	Desveaux et al., 2004
*Arabidopsis thaliana*	*oeWHIRLY1*	No phenotype	Hypersensitive to ABA	Isemer et al., 2012a
*Arabidopsis thaliana*	*oeWHIRLY2*	Accelerated senescence, reduced pollen growth		Maréchal et al., 2008; Cai et al., 2015
*Arabidopsis thaliana*	*why2 T-DNA mutant*	Reduced germination		Golin et al., 2020
*Hordeum vulgare*	RNAi *WHIRLY1* knockdown	Delay of chloroplast development and senescence	Reduced resistance to high light, enhanced drought resistance, reduced resistance toward powdery mildew (Hensel et al., unpublished)	Janack et al., 2016; Kucharewicz et al., 2017; Swida-Barteczka et al., 2018; Krupinska et al., 2019
*Hordeum vulgare*	oe*WHIRLY1*	Delay of senescence	Enhanced resistance toward powdery mildew	Krupinska, unpublished
*Manihot esculenta*	oe*WHIRLY1-3*		Enhanced resistance toward cassava bacterial blight	Liu et al., 2018
*Manihot esculenta*	Virus-induced silencing of *WHIRLY1-3*		Higher sensitivity toward cassava bacterial blight	Liu et al., 2018
*Manihot esculenta*	Silencing of *WHIRLY1* expression		Reduced drought resistance	Yan et al., 2020
*Nicotiana tabacum*	oe*SlWHIRLY2*		Enhanced resistance toward drought and *Pseudomonas solanacearum*	Zhao et al., 2018
*Solanum lycopersicum*	o*eSlWHIRLY1*		Enhanced chilling tolerance by upregulated expression of *RBCS1*	Zhuang et al., 2019, 2020b
*Solanum lycopersicum*	o*eSlWHIRLY1*		Enhanced thermotolerance by regulation of HSP21.5A expression	Zhuang et al., 2020a
*Solanum lycopersicum*	RNAi *SlWHIRLY1*		Reduced thermotolerance	Zhuang et al., 2020a
*Solanum lycopersicum*	RNAi *SlWHIRLY1*		Enhanced chilling sensitivity	Zhuang et al., 2019
*Solanum lycopersicum*	RNAi *SlWHIRLY2*		Reduced drought resistance	Meng et al., 2020
*Zea mays*	*why1* Transposon mutant	Inhibition of chloroplast development		Prikryl et al., 2008

In contrast, *ZmWHIRLY1* transposon mutants of *Zea mays* have ivory or albino leaves and die after developing three or four leaves (Prikryl et al., 2008). Biochemical analyses revealed that impaired ribosome formation and disturbed splicing might cause impaired chloroplast development (Prikryl et al., 2008). Following the positive impact of WHIRLY1 on chloroplast development in maize, a barley line with a low abundance of WHIRLY1 was shown to be delayed in the formation of ribosomes and in chloroplast development (Krupinska et al., 2019).

In many plants, including Arabidopsis, mutations with such a profound impact on plastid ribosome formation preventing chloroplast development would cause embryo lethality (Belcher et al., 2015). However, in cereals with large grain reserves supporting heterotrophic growth, the molecular defects of such mutations can be studied. Intriguingly, *ZmWHIRLY1* was shown to be identical with the gene *EMBRYO DEFECTIVE 16*, whose mutation caused embryo lethality (Zhang et al., 2013). The authors showed that the consequences of the *ZmWHIRLY1* mutation depend on the genetic background of the different maize varieties. A cross between *Zmwhy1* and *Zmemb16* resulted in defective embryos and albino seedlings in the F1 progeny (Zhang et al., 2013). The authors hypothesized that the negative impact of WHIRLY1 deficiency on embryogenesis is related to impaired plastid translation. In some genetic backgrounds of maize, embryo lethality usually resulting from impaired plastid translation might be suppressed.

It is possible that Arabidopsis mutants neither show a chloroplast development phenotype nor an embryo development phenotype because another WHIRLY protein might replace the mutated one. Indeed, due to the dual targeting of AtWHIRLY3 to chloroplasts and mitochondria (Golin et al., 2020), it is likely that WHIRLY3 can replace both WHIRLY1 and WHIRLY2. The *AtWHIRLY3* gene is apparently indispensable because there is no report on a knockout mutant of *AtWHIRLY3*. The truncated WHIRLY3 in the TILLING *why3* mutant is terminated after amino acid 98 and therefore still contains the DNA-binding motif KGKAAL (Supplementary Figure 2). The “*why1why3*” double mutant for *AtWHIRLY1* and *AtWHIRLY3*, which has been frequently used in studies on the WHIRLY proteins, was prepared by crossing the T-DNA insertion mutant *why1-1* and the tilling mutant *why3* from the Arabidopsis TILLING project (ATP; Till et al., 2003; Maréchal et al., 2009) and should be precisely termed *why1tilwhy3*.

So far, there is no evidence for a mutant in which all WHIRLY genes are knocked out in any plant species. Targeted mutation by CRISPR/Cas9 technology did not succeed in the mutation of the *AtWHIRLY3* gene while mutation of *AtWHIRLY1* and *AtWHIRLY2* was possible (Hensel, unpublished). These mutants show no obvious chloroplast development defects (Krupinska et al., unpublished). Considering that single mutants and even Arabidopsis double mutants can still perform essential functions shared by all WHIRLY proteins, it is reasonable to expect that all WHIRLY proteins can locate to all three DNA containing compartments of the cell.

Chloroplasts, besides mitochondria, are the powerhouses of plant cells whose operation needs to be tightly controlled. It is well-known that disturbances in chloroplast development have consequences for retrograde signaling altering nuclear gene expression during all phases of plant development (Pfannschmidt and Munné-Bosch, 2013; Chan et al., 2016). Due to the high susceptibility of the photosynthetic apparatus to environmental cues, coordination of plastidic and nuclear activities is of pivotal importance. During leaf senescence, chloroplasts are dismantled, and nutrients must be efficiently remobilized (Pfannschmidt and Munné-Bosch, 2013; Krieger-Liszkay et al., 2019). Senescence processes may be prematurely induced and accelerated by environmental factors including light, reduced water supply, and hormones known to accumulate during stress and senescence, that is, abscisic acid, jasmonic acid, and salicylic acid. While light accelerates senescence in barley wild-type plants, the RNAi-mediated *WHIRLY1* knockdown plants are compromised in response to light, albeit the senescence-associated hormones have high levels in the leaves of these plants (Kucharewicz et al., 2017). This result is in accordance with the observation that the *why1* mutant of Arabidopsis is insensitive to abscisic acid (Isemer et al., 2012a). Taken together, both studies showed that WHIRLY1 enhances the responsiveness to ABA in both dicot and monocot plants. With regard to this feature shared by WHIRLY1 of monocots and dicots, it is surprising that senescence is promoted in the *why1* mutant of *A. thaliana* (Miao et al., 2013). The rapid senescence in the latter has been assigned to the repressing effect of AtWHIRLY1 on the expression of *WRKY33* and *WRKY53* encoding senescence promoting transcription factors (Miao et al., 2013).

While WHIRLY1 is involved in chloroplast biogenesis and senescence, WHIRLY2 has been reported to control germination. Although the development of vegetative parts is not affected in the mature *Atwhy2* T-DNA insertion mutant, seed germination is compromised in the mutant (Golin et al., 2020). In line with this observation, the expression level of *AtWHIRLY2* in seeds is higher than that of *AtWHIRLY3*, whereas the expression of the two genes does not show differences in the vegetative parts of mature wild-type plants. The differential expression of *AtWHIRLY2* and *AtWHIRLY3* predetermines the possibility of a functional replacement of AtWHIRLY2 by AtWHIRLY3 (Golin et al., 2020).

## Whirly Proteins Are Architectural Organelle Nucleoid-Associated Proteins

After HvWHIRLY1 was shown to be located in chloroplasts and the nucleus of the same cell (Grabowski et al., 2008), it had been suggested that transcription factors, such as WHIRLIES, might be stored in chloroplasts or mitochondria. They were proposed to rapidly translocate to the nucleus upon certain stimuli (Krause and Krupinska, 2009). However, analyses of organelle structure and nucleoid morphology in plants with reduced abundance of WHIRLY proteins clearly showed that the proteins also have essential organellar functions related to their binding to ssDNA and RNA (Prikryl et al., 2008; Melonek et al., 2010).

WHIRLY2 promotes DNA compaction in Arabidopsis mitochondria, as evident in transmission electron microscopic images of the *why2* mutant mitochondria containing largely unpacked DNA (Golin et al., 2020). Furthermore, WHIRLY2 was found together with another abundant ssDNA-binding protein (ODB1 = RAD52) in large DNA- and RNA-containing complexes likely deriving from nucleoids (Table 3). Both proteins were shown to interact *via* mitochondrial DNA (Janicka et al., 2012). The study’s authors discussed that WHIRLY2, together with ODB1, cover the mitochondrial DNA (mtDNA) at sites where ssDNA is formed during the division of the organelles. It remained unknown whether ODB1 and WHIRLY2 compete or cooperate in recruiting different factors to ssDNA regions (Janicka et al., 2012).

**Table 3:** Proteins co-immunoprecipitated, pulled-down, or co-purified with WHIRLIES in protein complexes of plastids or mitochondria.

	Full name and synonymous names	WHIRLIES	Organelle protein complex	Function	References
BCCP1	Biotin carboxyl carrier protein 1 of acetyl-CoA carboxylase	WHIRLY3	Nucleoid	Fatty acid biosynthesis	Phinney and Thelen, 2005
CLPC1	CLP protease chaperon 1	WHIRLY3	Nucleoid	Proteolysis stress	Zheng et al., 2002; Olinares et al., 2011
CRS1	Chloroplast RNA Splicing 1	WHIRLY1	Ribonucleoprotein complex	Group IIA intron splicing factor	Till et al., 2001; Prikryl et al., 2008
2CPA	2-Cys-Peroxiredoxin A	WHIRLY1 WHIRLY3		Redox sensing and redox regulation	Liebthal et al., 2020; Telman et al., 2020
FIB1a	Fibrillin 1a	WHIRLY3	Network around plastoglobuli, interaction with fibrillin 1b indicative of oligomerization	Involved in partitioning of proteins in plastids protection against photodamage, stress resistance	Singh and McNellis, 2011; Gamez-Arjona et al., 2014
FIB4	Fibrillin 4	WHIRLY3	Interaction with plastoglobules, LHC, photosystem II, thylakoids	Development of plastoglobules, resistance to multiple stresses	Singh et al., 2010
pTAC4	Plastid transcriptionally active chromosome 4 VIPP, vesicle inducing plastid protein	WHIRLY3	Nucleoid/TAC	Chloroplast development	Kroll et al., 2001
PPR4	Plastid pentatricopeptide repeat 4	WHIRLY3	Ribonucleoprotein complex	*rps12* trans-splicing	Xu et al., 2019
LHCA1	Light-Harvesting complex of photosystem I, subunit 1	WHIRLY1	Photosystem I	Photosynthesis, light adaptation	Huang et al., 2017
LHCB5	Light-Harvesting complex of photosystem II, subunit 5 CP26	WHIRLY3	Photosystem II	Photosynthesis, tolerance to drought, oxidative stress protection	Xu et al., 2012; Chen et al., 2018
ODB1	Organelle DNA-binding protein 1, RAD52-1	WHIRLY2	Large nucleoprotein complexes, likely nucleoids	Recombination and repair of mitochondrial DNA	Janicka et al., 2012
PetA	Cytochrome f	WHIRLY3	Cytochrome b6f complex	Photosynthesis, electron transport	
PRPS5	Plastid ribosomal protein S5, EMB3113	WHIRLY3	Essential component of plastid ribosome	16S rRNA processing, translation cold stress tolerance embryo development	Bryant et al., 2011; Zhang et al., 2016
PRPS3	Plastid ribosomal protein S3	WHIRLY3	Essential component of plastid ribosome	Translation affects leaf shape	Fleischmann et al., 2011
RECA	Recombinase A	WHIRLY2		Mitochondrial DNA recombination	Meng et al., 2020
RNaseH	Ribonuclease H	WHIRLY1 WHIRLY3	DNA gyrase complex	Maintenance of ptDNA, removal of R-loops	Yang et al., 2017

In chloroplasts, WHIRLIES have been identified as significant components of the transcriptionally active chromosome (TAC; Pfalz et al., 2006; Melonek et al., 2010; Majeran et al., 2012). Therefore, WHIRLY1 and WHIRLY3 are also called pTAC1 and pTAC11, respectively. TAC is used as a term used for nucleoids when biochemical properties rather than structural aspects are in the focus of the investigations (Krause and Krupinska, 2017). Nucleoids were proposed to be multifunctional platforms serving various DNA-associated processes, that is, replication, repair/recombination, and transcription (Sakai et al., 2004; Kobayashi et al., 2016). Moreover, their complex proteomes suggest that they serve as a docking station for proteins with key functions in metabolic processes and signaling (Melonek et al., 2016).

**Figure 3:**
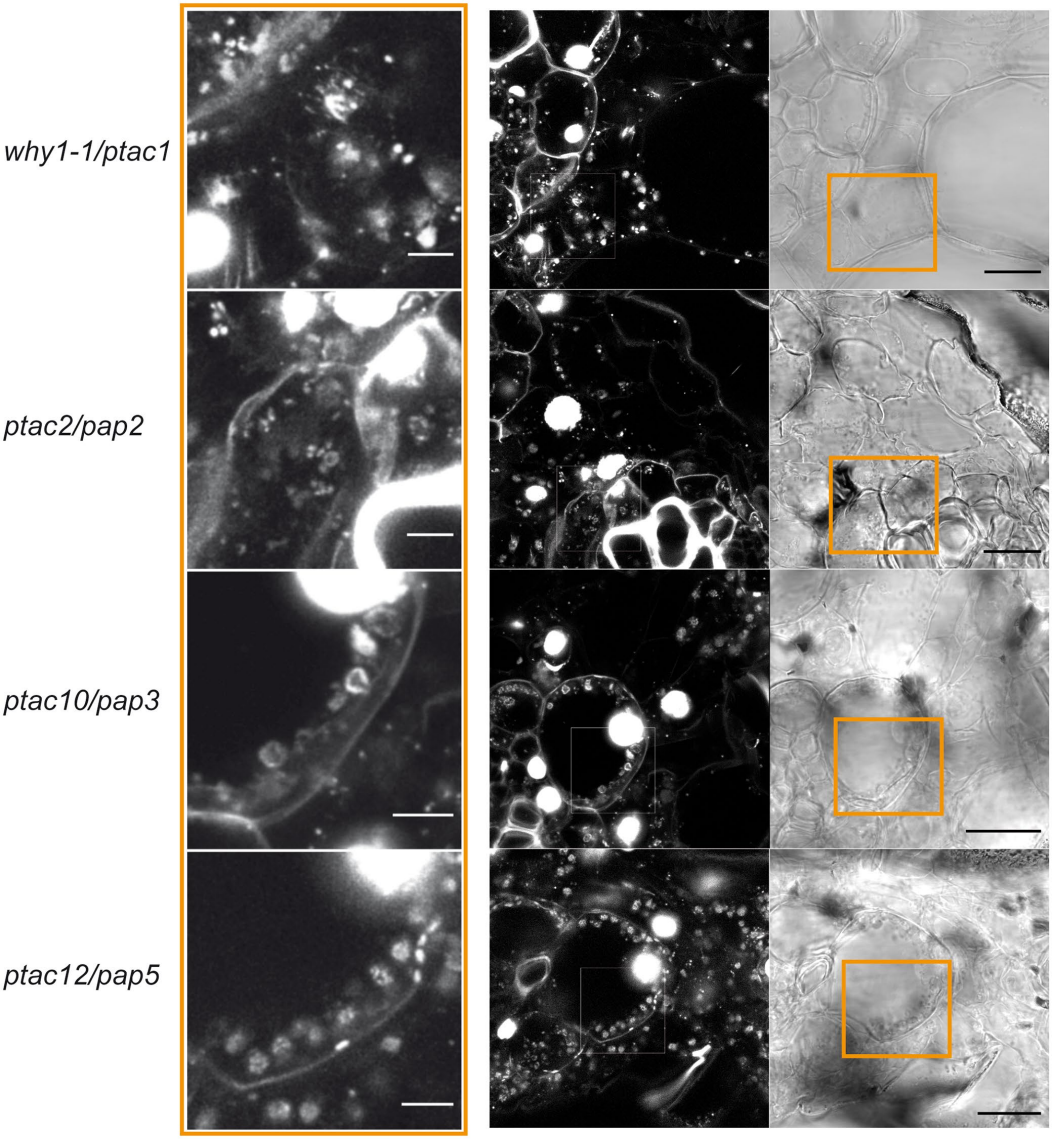
Morphology of nucleoids in sections from albino leaves of maize transposon mutants. The *Zmwhy1*-1 mutant (Prikryl et al., 2008) is compared with selected mutants lacking PEP-associated proteins (PAP; Williams-Carrier et al., 2014). All proteins are transcriptionally active chromosomes (TAC) components and hence have pTAC names. DNA was stained by SYBR GREEN as described (Krupinska et al., 2014b). Bars in low magnification images represent 20 μm, in the higher magnifications on the left, bars represent 5 μm. Frames in the transmission images on the right indicate the sections shown in the high magnification images on the left.

While WHIRLY1 and WHIRLY3 in Arabidopsis plastids have no noticeable impact on nucleoid morphology, chloroplasts of transgenic barley plants with an RNAi-mediated knockdown of *HvWHIRLY1* contain unpacked plastid DNA (ptDNA) besides a few regularly packed nucleoids (Krupinska et al., 2014b). The staining of DNA in the ivory leaves of the transposon insertion mutant *Zmwhy1-1* revealed that the ptDNA is detectable in patches without a prominent characteristic nucleoid structure (Prikryl et al., 2008; Figure 3). For comparison, DNA was stained in ivory leaves of other non-photosynthetic maize mutants with transposons inserted in genes encoding other TAC proteins, that is, pTAC2 (PAP2), pTAC10 (PAP3), and pTAC12 (PAP5; Williams-Carrier et al., 2014). In contrast to the morphologically of unorganized nucleoids of the *Zmwhy1-1* mutant, nucleoids of the *pap* mutants are discernible as distinct punctate speckles that form structures resembling a necklace of pearls or rings associated with the inner envelope of plastids. Similar structures have also been observed in undifferentiated plastids of wild-type meristematic cells and albino leaf sections of the barley mutant *albostrians* (Powikrowska et al., 2014).

WHIRLY1 proteins of dicot species and WHIRLY2 proteins of monocot and dicot species did not affect nucleoid compaction in *Escherichia coli* (Oetke et al., 2022). In accordance with this finding, a mutation of the conserved second lysine of the KGKAAL motif did not affect the compactness of nucleoids in *E. coli.* Surprisingly, the PRAPP motif that has been identified in monocot WHIRLY1 proteins promoted compaction in *E. coli* but not in the chloroplasts of transgenic Arabidopsis plants with PRAPP inserted into *AtWHIRLY1* (Oetke et al., 2022). Taken together, there is no evidence for compaction of plastid nucleoids by WHIRLY proteins in Arabidopsis. Alternatively, plastid nucleoid compaction might be mediated by SWIB proteins in Arabidopsis (Melonek et al., 2012) and by sulfite reductase containing a DNA compacting motif in tobacco (Cannon et al., 1999).

It is possible that the reduced compactness of nucleoids as observed in barley *WHIRLY1* knockdown plants (Krupinska et al., 2014b) and the maize *why1* mutants (Figure 3), but not in the *why* mutants of Arabidopsis (Oetke et al., 2022), has negative consequences for chloroplast development by impairing ribosome formation. Following the idea that nucleoids serve as platforms for the formation of ribosomes (Bohne, 2014), several components of ribosomes have been detected in the nucleoid proteomes (Melonek et al., 2016). Hence, it is conceivable that the deficiency in ribosome formation as observed in maize plastids is a consequence of uncompacted nucleoid DNA (Figure 3). A coincidence of unorganized nucleoids and reduced levels of plastid ribosomal RNAs indicating a reduced ribosome content was also observed in the barley *WHIRLY1*-RNAi plants with a low abundance of WHIRLY1 (Krupinska et al., 2014b, 2019).

Concerning its nucleoid compacting activity in monocot chloroplasts, WHIRLY1 belongs to the group of abundant architectural nucleoid-associated proteins (NAPs) that in bacteria have also been termed histone-like proteins (HPL; Luijsterburg et al., 2006, 2008; Dillon and Dorman, 2010). Bacterial NAPs with high abundance bind to DNA with low specificity and protect DNA by coating/compacting in response to environmental stress (Holowka and Zakrzewska-Czerwinska, 2020). A prominent example is the HU protein which binds sequence independently to DNA and regulates global gene expression by nucleoid remodeling (Remesh et al., 2020). Indeed, several parallels between the properties of WHIRLY proteins and the bacterial HU proteins are apparent and have been discussed by Prikryl et al. (2008): both proteins bind preferentially to ssDNA in addition to RNA and are involved in recombination and repair of DNA.

The structure of the WHIRLY 24-mer shares similarity with that of the oligomeric bacterial protein Dps (DNA-binding protein of starved cells), whose gene is highly expressed during stress and which is responsible for the compaction of nucleoids in the stationary phase and during stress (Dame et al., 2020; Holowka and Zakrzewska-Czerwinska, 2020). Both, Dps and WHIRLY proteins bind non-specifically to DNA *via* lysine residues through a cooperative mechanism in which supramolecular nucleoprotein complexes are formed (Wolf et al., 1999; Cappadocia et al., 2012). Protection of DNA during stress is achieved by NAP-mediated coating and condensing of the nucleoid, thereby creating a selective physical barrier separating the DNA from the surrounding matrix (Holowka and Zakrzewska-Czerwinska, 2020). Experimentally, it has been demonstrated that the condensation of nucleoids by Dps does not affect the access of RNA polymerase (RNAP) to DNA while other proteins are prevented from interacting with DNA (Janissen et al., 2018; Abbondanzieri and Meyer, 2019). Already as early as 1984, it had been suggested that nucleoids are a membrane-less microcompartment formed by phase separation (Valkenburg and Woldringh, 1984; Odijk, 1998). It is self-evident that abundant NAPs, such as HU, Dps, and likely WHIRLIES, are the building blocks of nucleoids. A putative cage-forming activity of WHIRLY1 in plastid nucleoids is in accordance with peripheral localization of WHIRLY1 in the nucleoids (Melonek et al., 2010; Krupinska et al., 2013).

## Whirlies Impact Photosynthesis and Respiration

Photosynthesis in chloroplasts and respiration in mitochondria provide the energy for plant development and growth. It has been reported that the expression levels of *AtWHIRLY1* and *AtWHIRLY3* increased after the application of inhibitors affecting these processes (Karpinska et al., 2017), indicating that WHIRLY proteins are required for efficient photosynthesis and respiration and that a reduction of WHIRLIES could reduce energy production. Furthermore, *WHIRLY2* expression was also shown to significantly increase after treating Arabidopsis seedlings with the translation inhibitor spectinomycin, preventing chloroplast development (Börner and Krupinska, unpublished results).

**Figure 4:**
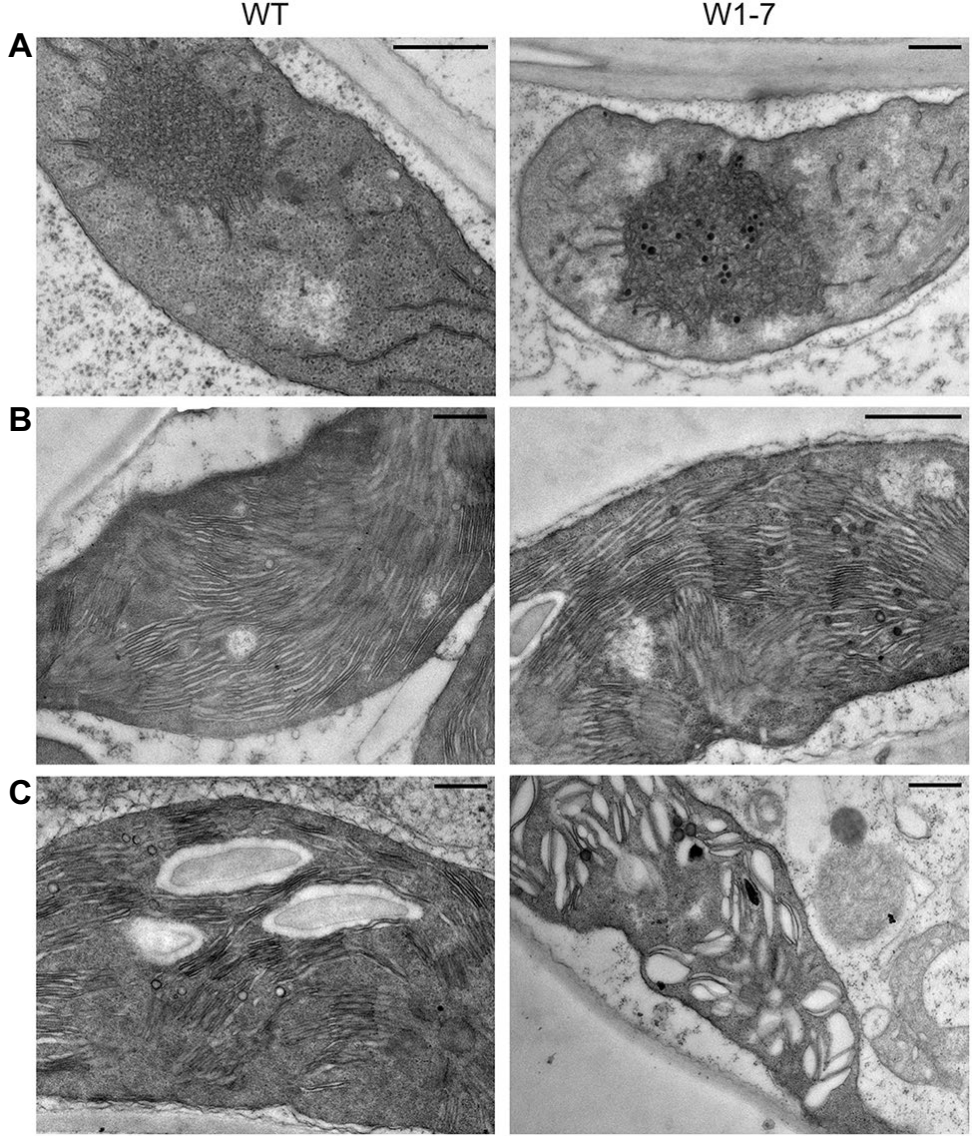
Impact of WHIRLY1 on the structure of prolamellar bodies and prothylakoids of etioplasts **(A)** and thylakoids of barley chloroplasts **(B,C)**. Ultrathin sections were prepared from primary foliage leaves of RNAi-W1-7 and wild-type plants. For analysis of etioplast structure, plants were grown in darkness for 8 days. For studies of chloroplasts, plants were grown in continuous light of either 120 **(B)** or 320 μmol m^−2^ s^−1^ **(C)**. Ultrastructural analyses were performed as described (Golin et al., 2020). Bars represent 500 nm.

The characterization of the WHIRLY1 deficient barley plants revealed that the delay in chloroplast development coincides with disturbances in the structure of prolamellar bodies and thylakoids (Figure 4A). Prolamellar bodies are clearly discernible in etioplasts of WHIRLY1 deficient plants grown in darkness. However, the organization of the tubular structures is less regular than in wild-type etioplasts. In chloroplasts of the WHIRLY1 deficient plants, the organization of thylakoid membranes appears to be less regular, with grana stacks having more various heights than in wild-type chloroplasts (Saeid-Nia et al., 2022). While during growth in a daily light–dark cycle, the major part of the thylakoids stays stacked even at high irradiance (Saeid-Nia and Krupinska, unpublished), during continuous illumination at high irradiance, thylakoids of WHIRLY deficient chloroplasts get swollen (Figure 4B). This swelling indicates that the light stress effects accumulating during continuous illumination can be minimized by WHIRLY1.

The structural abnormalities of the thylakoid membranes observed in WHIRLY1 deficient barley plants indicate that WHIRLY1 is required for an accurate assembly of the photosynthetic apparatus. During the development of primary foliage leaves of barley with reduced abundance of WHIRLY1, chlorophyll content and the efficiency of photosystem II measured by chlorophyll fluorescence increased much slower than in wild-type leaves (Krupinska et al., 2019). When the plants were grown in continuous light of different irradiances, chlorophyll levels were severely reduced in the WHIRLY1 deficient plants grown at high irradiance. The irradiance dependent decline in the chlorophyll content coincided with the production of reactive oxygen species (ROS) indicating that the photosynthetic apparatus cannot be adjusted to the prevailing light conditions when the abundance of WHIRLY1 is low (Swida-Barteczka et al., 2018). Additional investigations on light acclimation revealed that the WHIRLY1 deficient barley plants show neither the typical high light-induced increase in photosynthesis nor leaf thickness as observed in wild-type plants (Saeid Nia et al., 2022). Basically, the results of these studies indicate that the WHIRLY1 deficient plants are either compromised in sensing light quantity or in reacting to a change in light intensity. Considering that the WHIRLY1 deficient plants have a lower chlorophyll a/b ratio, they are rather compromised in the measurement of light intensity, than in responding to light intensity by changes in the composition of the photosynthetic apparatus (Krupinska et al., 2019).

Curiously, WHIRLY1 deficient barley plants were reported to maintain higher assimilation rates under limiting nitrogen than wild-type plants (Comadira et al., 2015). However, they are compromised in photosynthetic performance when grown in soil (Krupinska et al., 2019; Saeid Nia et al., 2022). This finding suggests that WHIRLY1 is not only important for acclimation to light, but also to nutrient deficiency. Accordingly, the expression of *HvWHIRLY1* is upregulated in response to nitrogen deficiency (Comadira et al., 2015).

So far, the impact of WHIRLY2 on the structure and function of mitochondria could be analyzed in the *why2* T-DNA insertion mutant of *A. thaliana* (Golin et al., 2020) and tomato plants (Meng et al., 2020). Mitochondria of the Arabidopsis mutant have a lower number of cristae and a low electron density as analyzed by transmission electron microscopy. The structural changes correlate with reduced respiration activity (Golin et al., 2020). Intriguingly, the overexpression of *AtWHIRLY2* negatively affected the morphology and functionality of mitochondria (Maréchal et al., 2008). Overexpression of *AtWHIRLY2* under control of a pollen-specific promoter led to slower pollen tube growth due to an increase in ROS and a decrease in ATP production (Cai et al., 2015). Tomato plants with a reduced accumulation of WHIRLY2 were reported to have lower expression levels of mitochondrial genes encoding components of respiratory complexes, lower alternative oxidase activity, and enhanced ROS levels (Meng et al., 2020). It seems that either a reduced or an increased abundance of WHIRLY2 has detrimental effects on mitochondrial structure and function. An optimal abundance of WHIRLY2 in mitochondria seems to be critical for both structure and function, that is, respiration and ATP production.

## Organelle Proteins Interacting With Whirlies

To learn about the multiple functions of WHIRLIES, it is helpful to know more about their interactions with other proteins. About half of the proteins experimentally shown to interact with one or more WHIRLIES (Supplementary Table 1) are organellar proteins, with the majority of these being active in the plastids (Table 3). Concerning the localization of WHIRLIES in organelle nucleoids, the identification of further nucleoid-associated proteins (NAPs) as interacting proteins is foreseeable. Surprisingly, however, only one of the 12 proteins found to co-immunoprecipitate with an AtWHIRLY3-specific antibody (Isemer, 2013) is a NAP, that is, pTAC4. The protein that has also been named vesicle inducing plastid protein (VIPP1), is essential for the formation of thylakoids during chloroplast development (Kroll et al., 2001).

Furthermore, WHIRLIES were found to interact with the two ribosomal proteins, PRPS5 and PRPS3, also located in nucleoids (Melonek et al., 2016). Besides, WHIRLY3 interacts with BCCP1, a subunit of acetyl CoA carboxylase (ACCase) that also belong to the subgroup of “unexpected” nucleoid-associated proteins that do not have a known function associated with DNA (Melonek et al., 2016). However, in the protein repertoire of a fraction enriched in ACCase from chloroplasts, among 179 proteins, 35 proteins were predicted to be nucleoid-associated proteins (Phinney and Thelen, 2005), indicating a close link between nucleoids and biosynthetic activities in chloroplasts (Melonek et al., 2016). Another “unexpected” nucleoid protein that interacts with WHIRLY3 is the chaperone of the CLP protease, that is, CLPC1. Furthermore, WHIRLIES in chloroplasts were also found to interact with RNA-binding proteins, that is, CRS1, PPR4. WHIRLY1/3 proteins were identified by mass spectrometry in complexes that co-immunoprecipitated with Arabidopsis chloroplast RNase H1 (Yang et al., 2017). The interaction with RNase H1 was mediated by DNA and was proposed to be essential for protecting DNA by WHIRLIES (Wang et al., 2021).

With regard to the impact of WHIRLIES on chloroplast structure (see above), it is not surprising that two fibrillins, FIB1a and FIB4, are among the interacting proteins found by co-immunoprecipitation with a WHIRLY3 specific antibody. Fibrillins form a network around plastoglobules and partition plastid proteins (Singh and McNellis, 2011; Gamez-Arjona et al., 2014; Torres-Romero et al., 2022).

Furthermore, WHIRLIES in chloroplasts were found to interact with several proteins of the photosynthetic apparatus, that is, LHCA1 that has been co-immunoprecipitated with AtWHIRLY1 (Huang et al., 2017) and LHCB5 as well as cytochrome f (PetA) that have been co-immunoprecipitated with AtWHIRLY3 (Isemer, 2013). These interactions are in accordance with the impact of HvWHIRLY1 on thylakoid membrane structure (Figures 4B,C) and photosynthetic function (Krupinska et al., 2019). The photosynthetic apparatus undergoes redox changes in response to the environment that are signaled to the nucleus (Fey et al., 2005). The interaction of WHIRLY1 with 2-cysteine peroxiredoxin (2CPA) might be linked to redox sensing and signaling (Liebthal et al., 2020).

In mitochondria of tomato, WHIRLY2 was found to interact with RECA2, a subunit of a mitochondrial recombinase (Meng et al., 2020). Arabidopsis WHIRLY2 has been purified with the ssDNA-binding DNA-binding protein ODB1 (RAD52-1), which has a dual localization in the nucleus (Janicka et al., 2012). Both interactions might be necessary for maintaining and repairing mitochondrial DNA.

Two proteins that were shown to interact with WHIRLY3 in yeast two-hybrid assays are enzymes: one is an isomer of mitochondrial cysteine synthase, and the second is aconitase 1 (Isemer, 2013), which belongs to the 4Fe-4S cluster enzymes and catalyzes the isomerization of citrate to isocitrate in mitochondria (Przybyla-Toscano et al., 2021). For rice aconitase, a role in iron sensing and regulation of Fe deficiency-inducible genes has been reported (Senoura et al., 2020).

## Impact of Whirlies on Organelle DNA-Associated Activities

Concerning their abundance in nucleoids and their binding affinities to DNA and RNA as well as the reported interactions with nucleoid-associated proteins (Table 3), WHIRLIES have an impact on the multiple processes associated with organelle DNA, that is, replication, recombination, and maintenance of genome stability, transcription and associated posttranscriptional processes. The effects of WHIRLIES on these processes were mainly investigated with the Arabidopsis *why1tilwhy3* mutant, maize *why1* mutants, and barley plants with a knockdown of *WHIRLY*.

### Replication

Barley plants with an RNAi-mediated knockdown of *WHIRLY1* contained higher levels of ptDNA in their leaves (Krupinska et al., 2014b). This result coincided with a higher expression of plastid–mitochondria targeted plant organelle DNA polymerase (POP; Krupinska et al., 2014b), suggesting that WHIRLY1 might have a repressing effect on the expression of the POP encoding gene. In contrast, ptDNA levels are not altered in *why1* mutants of maize (Prikryl et al., 2008) and Arabidopsis (Maréchal et al., 2009).

Curiously, in pollen of Arabidopsis, the level of mtDNA was found to correlate with the abundance of AtWHIRLY2. Overexpression of *AtWHIRLY2* under control of the vegetative cell-specific promoter *Lat52* increased the mtDNA level by 10 times. In these plants, pollen were shown to have enhanced ROS levels and less ATP, coinciding with slower growth of pollen tubes (Cai et al., 2015).

### Transcription

Transcription in chloroplasts is mediated by two RNA polymerases, the bacterial type plastid-encoded RNA polymerase PEP (plastid-encoded polymerase) and a nucleus-encoded polymerase resembling those of T-phages (RPOT) that is also termed NEP (nucleus-encoded polymerase; Hess and Börner, 1999b; Börner et al., 2015). Principally, both enzymes transcribe all genes, but from different promoters and with different efficiencies. While NEP provides the basic level of transcription in all types of plastids (Hess and Börner, 1999a) or chloroplasts at night (Ono et al., 2020), PEP is required for the dramatic increase in transcriptional activity required for chloroplast development (Baumgartner et al., 1989; Börner et al., 2015). In a very recent study, it has been shown that binding of PEP to DNA is reduced in chloroplasts of a triple mutant obtained from *why1tilwhy3* and a mutant lacking its interacting protein RNase H1C (Table 3; Wang et al., 2021). In a preceding paper, the interaction of WHIRLY proteins with RNase H1 had been suggested to promote transcription (Yang et al., 2017).

During chilling, tomato WHIRLY1 was found to bind to a specific motif (GTTACCCT) in the promoter of the *psbA* gene, which is mainly transcribed by PEP. This motif has also been detected in pepper, another chilling sensitive plant, but not in Arabidopsis, maize, and rice (Zhuang et al., 2019). Because WHIRLY1 was found neither to interact with PEP nor NEP, the significance of the sequence-specific binding remained unknown.

Barley plants with an RNAi-mediated knockdown of *WHIRLY1* showed reduced levels of PEP-dependent transcripts but high levels of NEP-dependent transcripts, such as *rpoB/C* and *clpP* encoding the subunits of PEP and the catalytic subunit of the CLP protease (Krupinska et al., 2019). This decrease in the ratio of PEP- and NEP-derived transcripts indicates an impaired function of PEP coinciding with retardation of chloroplast development. The reduced activity of PEP which requires supercoiled rather than relaxed templates (Zaitlin et al., 1989) in the WHIRLY1 deficient barley chloroplasts may be a consequence of the reduced compaction of plastid nucleoids (Krupinska et al., 2014b).

Underrepresented PEP-derived transcripts include ribosomal RNAs (Börner et al., 2015). A reduction in ribosomal RNA is shared by the barley RNAi-WHIRLY1 plants, the *Zmwhy1* mutants, and the Arabidopsis double mutant *why1tilwhy3*, as reported recently (Wang et al., 2021). Maréchal et al. (2009) did, however, not observe a reduced level of plastid ribosomal RNA in the same *why1tilwhy3* mutant and concluded that the variegation observed in a part of the mutant progeny might have another reason. Intriguingly, in all cases, the level of 23S rRNA is more affected than the level of 16S rRNA. This altered expression might be explained by replication–transcription conflicts occurring at a putative replication origin located near the 23S rDNA in the region of the inverted repeat (IR; Takeda et al., 1992).

### RNA Splicing

In barley (Melonek et al., 2010) as well as in algae and liverwort (Kobayashi et al., 2016), WHIRLIES were shown to have an additional extra-nucleoid localization that might be related to their association with RNA derived from intron-containing genes, their interaction with the splicing factors PPR4 and CRS1 (Table 3) and their positive impact on splicing of primary transcripts (Prikryl et al., 2008; Melonek et al., 2010). It remains an open question whether the inefficient splicing of plastid transcripts in WHIRLY1 deficient plants is a direct consequence of WHIRLY1 deficiency or is caused by a disturbed formation of plastid-encoded RNA polymerase (PEP). Indeed, in transplastomic tobacco plants lacking a functional PEP, RNA processing is altered (Legen et al., 2002), indicating that the origin of transcripts determines their processing. Intriguingly, accumulation and processing of mitochondrial transcript is also altered in Arabidopsis plants overexpressing the *AtWHIRLY2* gene (Maréchal et al., 2008).

### Maintenance of Organelle Genome Stability

Organelle genomes have to be faithfully repaired to ensure the efficient functioning of organelles and overall plant performance (Maréchal and Brisson, 2010). To maintain the integrity of the genome, chloroplasts and mitochondria have efficient DNA recombination surveillance machineries (Maréchal and Brisson, 2010; Kühn and Gualberto, 2012). The involvement of plastid WHIRLIES in the repair of DNA by homologous recombination has been first reported for the double mutant *why1tilwhy3* showing a variegation phenotype in a portion of the mutant progeny (about 5%; Maréchal et al., 2009). Variegated leaf parts of the double mutant accumulated aberrant ptDNA resulting from microhomology-mediated illegitimate recombination (MMIR; Maréchal et al., 2009). Maréchal et al. (2009) reported that the frequency of illegitimate recombination is also increased in the albino *why1*-*1* mutant of maize (Prikryl et al., 2008), albeit in this mutant, no large rearranged ptDNA molecules were detectable.

The variegated leaf phenotype of the *why1tilwhy3* was detectable with much higher abundance when the double mutant was combined with a mutant lacking the gene encoding organelle targeted DNA polymerase Ib (*polIb*; Zampini et al., 2015). A quadruple mutant lacking in addition RecA was unable to survive (Zampini et al., 2015). These investigations showed that WHIRLY proteins, DNA polymerase Ib and the RecA protein have synergistic effects on DNA stability.

In a recent study of the group of Normand Brisson, the double mutant *why1tilwhy3* was combined with the *sig6* mutant deficient in SIGMA 6 (SIG6), that is, a significant nucleus-encoded transcription factor for PEP (Di Giorgio et al., 2019). By characterization of the triple mutant and pharmacological treatments with the transcription inhibitor rifampicin, the authors identified transcription as a major source of ptDNA instability. Recently, it has been proposed that WHIRLY proteins promote the formation of RNA/DNA hybrids (R-loops) by recruiting RNAPs to DNA and that these R-loops serve in DNA repair by homologous recombination (Wang et al., 2021).

To fulfil their function in maintaining organelle genome stability, WHIRLIES require the DNA-binding motif KGKAAL. When the AtWHIRLY1 sequence mutated for the second lysine of the KGKAAL motif (K91A) was expressed under the control of the CaMV *35S* promoter in the *why1tilwhy3* mutant of Arabidopsis, the resulting plants were more sensitive toward the gyrase inhibitor ciprofloxacin (CIF) than control plants overexpressing the non-mutated WHIRLY1 (Cappadocia et al., 2012).

## Proteins Interacting With Whirlies in the Nucleus

Most interactions with nuclear proteins reported for all three Arabidopsis WHIRLIES have been shown by yeast two-hybrid assays, bimolecular fluorescence complementation, or by Cre reporter-mediated yeast two-hybrid coupled with next-generation sequencing (CrY2H) assays (Trigg et al., 2017; Supplementary Table 1). By the latter method, WHIRLIES were identified as components of the Arabidopsis transcription factor interactome (Trigg et al., 2017). Earlier, interactions of WHIRLIES with transcription factors were proposed to occur *via* the putative transactivation domains preceding the WHIRLY domain (Desveaux et al., 2005).

Several transcription factors interacting with WHIRLIES are involved in senescence and immune responses. One of the Arabidopsis transcription factors that interact with WHIRLY1 is WRKY53 (Trigg et al., 2017) whose gene expression during senescence is regulated by WHIRLY1 (Miao et al., 2013). Another member of the plant-specific family of WRKY transcription factors, that is, WRKY75, has been identified as WHIRLY interacting protein in cassava by yeast-2-hydrid assays and bimolecular fluorescence complementation (Liu et al., 2018). WHIRLY2 was found to interact with TGA1 (Trigg et al., 2017), belonging to the TGA motif transcription factors that have also been implicated in senescence and pathogen defense (Gatz, 2013). Both WRKY and TGA transcription factors might interact with WHIRLIES by binding to cis-elements that overlap with WHIRLY binding sequences (Table 1; Desveaux et al., 2005).

WHIRLY3 was found to interact with a member of the GATA transcription factor family member, that is, GATA14 belonging to the B-class of this large family (Behringer and Schwechheimer, 2015). GATA motifs are found in promoters of light-responsive genes. The B-GATA factors encoding genes are controlled by nitrogen availability, phytohormones, and light (Behringer and Schwechheimer, 2015).

Further plant-specific transcription factors interacting with WHIRLY2 or 3 are involved in growth and development, that is, the auxin response factor ARF19 involved in lateral root formation and response to phosphate starvation (Huang et al., 2018b; Lee et al., 2019), the two MADS-box class C transcription factors AGAMOUS-like 74 and 84 (AGL74 and AGL84; Castelan-Munoz et al., 2019) and WOX13 (He et al., 2019; Tvorogova et al., 2021). ARFs and WOXs are plant-specific families of transcription factors involved in regulation of developmental plasticity and responses to abiotic stress. WHIRLY2 was found to interact with the transcription factors AGL74 and AGL84 of the group of MADS-box factors (Table 4) involved in spatial and temporal plant developmental processes, such as flowering (Castelan-Munoz et al., 2019).

**Table 4:** Transcription factors interacting with WHIRLIES.

Transcription factor	Full names, synonymous names	Family, binding motif	WHIRLIES	Functional context	References
ARF19/IAA22 ARF11	Auxin response factor 19 Indole acetic acid inducible 22	Auxin response element	AtWHIRLY2 AtWHIRLY3	Ethylene responses, for example, root formation and response to phosphate starvation	Huang et al., 2018b
AGL74	AGAMOUS-LIKE 74	MADS-box class C CC(A/T)_6_GG	AtWHIRLY2	Spatial and temporal development/Flowering responses to stress	Castelan-Munoz et al., 2019
AGL84	AGAMOUS-LIKE 84	MADS-box class C CC(A/T)_6_GG	AtWHIRLY2	Plastid development and growth responses to stress	Castelan-Munoz et al., 2019
GATA14	Factor 14 binding to the cis-element GATA	Zn ion binding	AtWHIRLY3	Regulation of light-responsive genes	Reyes et al., 2004
TGA1	TGACG sequence-specific binding protein 1	bZIP basic leucine zipper	AtWHIRLY2	Redox-controlled regulation of systemic acquired resistance and different stress pathways	Gatz, 2013
WOX13	WUSCHEL-related homeobox 13, HB-4	Homeobox factor	AtWHIRLY3	Development, determination of cell fate, abiotic stress response	He et al., 2019
WRKY53	W-box binding factor 53 with WRKY motif	Group III of the WRKY transcription factors W-box	AtWHIRLY1	Leaf senescence and pathogen resistance	Miao et al., 2013, Huang et al., 2018a, Zentgraf and Doll, 2019
WRKY75	W-box binding factor 75 with WRKY motif	W-box	MeWHIRLY1, 2, 3	Disease resistance	Liu et al., 2018

Besides transcription factor interactions, other nucleus located proteins were found to interact with WHIRLIES. As early as in 2007, WHIRLY1 had been identified as a telomer binding protein. Later on, WHIRLY1 was demonstrated to physically interact with the catalytic subunit of telomerase (Majerska et al., 2017).

Furthermore, WHIRLIES were found to interact with components of signal transduction (Supplementary Table 1). WHIRLY1 interacts with CIPK14, that is, a CBL-interacting serine/threonine kinase 14 (Ren et al., 2017), likely involved in calcium signaling (Qin et al., 2008). WHIRLY2 was found to interact with ATARCA, a receptor of activated kinase C1A (RACK1) playing a role in numerous developmental processes and hormone responses (Guo et al., 2019).

## Functions of Nucleus Located Whirlies

By their affinity to ssDNA, nucleus located WHIRLIES are likely involved in all processes associated with the formation of ssDNA, such as transcription.

Brisson and co-workers proposed that WHIRLIES bind as tetramers to melted promoter regions to modulate the expression of target genes (Desveaux et al., 2002). By stabilizing melted promoter regions, WHIRLIES were proposed to enhance the activities of transcription factors interacting with them. More detailed information on the impact of WHIRLY binding to promoters on transcription has been reported for two senescence-associated genes regulated by WHIRLY1, that is, *AtWRKY53* and *HvS40*. WHIRLY1 binding to the promoter of Arabidopsis *WRKY53* has been shown by chromatin immunoprecipitation (Huang et al., 2018a). WHIRLY1 binding inhibits methylation and promotes acetylation of histone 3 (H3K9ac), coinciding with the recruitment of RNAPII and transcriptional activation of *WRKY53* during senescence (Huang et al., 2018a). In barley, WHIRLY1 was shown to bind to the promoter of the senescence-associated gene *S40* (Krupinska et al., 2014a). Furthermore, during drought-induced senescence WHIRLY1 affects histone modifications in the promoter and the coding sequence of *HvS40*. While euchromatic H3K9ac accumulated during drought stress in the wild type, no changes were observed in the *WHIRLY1* knockdown plants produced by RNA interference (Janack et al., 2016). The impaired change in chromatin structure coincided with a lack of stress-induced changes in the expression of multiple senescence and drought stress-related genes (Janack et al., 2016).

Taken together, the findings on transcription of the two senescence-associated genes in Arabidopsis and barley indicate that WHIRLY1 is involved in chromatin remodeling and might regulate the accessibility of DNA for transcription factors. Chromatin remodeling is also crucial for the maintenance of telomers (Lee and Cho, 2019). Interestingly, the AtWHIRLY1 deficient mutant plants show a steady increase in the length of telomers over generations (Yoo et al., 2007), being in accordance with the interaction of WHIRLY1 with telomerase (Supplementary Table 1).

It is known that chromatin remodeling also controls microRNA biogenesis at the transcriptional level (Choi et al., 2016). Promotion of microRNA biogenesis by WHIRLY1 (Swida-Barteczka et al., 2018) could be a further link between WHIRLIES and chromatin remodeling. The microRNAs play an essential role in the interactions of plants with the environment (Song et al., 2019) and serve in the attenuation of plant growth and development by targeting the mRNAs encoding transcription factors for degradation (Sunkar et al., 2012). Recently, microRNA biogenesis has been reported to be under the control of a retrograde signaling pathway from chloroplasts that involves tocopherols and 3′-phosphoadenosin 5′-phosphate, an inhibitor of exoribonucleases (Fang et al., 2019). So far, an interaction of WHIRLIES with proteins involved in microRNA biogenesis remains to be investigated. It will be interesting to elucidate whether WHRILIES affect microRNA biogenesis directly or *via* an impact on chromatin remodeling.

## Whirlies Are Involved in Transcriptional Networks Controlling Development and Stress Responses

Developmental and environmental cues control WHIRLY gene expression. Expression patterns might give information on the situations in which WHIRLIES might be necessary. In the promoters of the tomato WHIRLY genes, 13 types of putative transcription factor binding motifs have been identified, and the most dominant bind GATA and MYB transcription factors (Akbudak and Filiz, 2019). GATA factors play roles in light- and nitrate-dependent control of transcription (Reyes et al., 2004). Among them, GATA14 has been found to interact with WHIRLY3 (Table 4). MYB factors are implicated in abscisic acid (ABA) responses and play roles in development and in responses to abiotic and biotic stresses (Ambawat et al., 2013).

Expression of *HvWHIRLY1* does transiently increase during chloroplast development (Krupinska et al., 2014b) and declines during leaf senescence (Kucharewicz et al., 2017). In comparison, the expression level of *HvWHIRLY2* is high in meristematic tissue where WHIRLY2 it has been proposed to function as an epigenetic regulator (McCoy et al., 2021), declines during leaf development and increases during leaf senescence (Grabowski et al., 2008; Kucharewicz et al., 2017). Treatment of barley leaf segments with different hormones showed that ABA suppressed the expression of *WHIRLY1*, whereas expression of *WHIRLY2* increased by treatment with ABA (Grabowski et al., 2008). Treatment with methyl jasmonate had inverse effects on the expression of the two genes (Grabowski et al., 2008).

While the two WHIRLY genes in barley responded differently during development and toward treatment with stress-associated hormones, both WHIRLY genes of tomato are likewise upregulated during drought and salt stress (Akbudak and Filiz, 2019). Furthermore, *WHIRLY1* of tomato was observed to have higher expression during chilling (Zhuang et al., 2019). The three *WHIRLY* genes of cassava (*Manihot esculentum*, *Me*) are upregulated by the bacterial *flg22* elicitor and treatment with Xanthomonas (Liu et al., 2018). Expression of all five *WHIRLIES* genes in strawberry was shown to be downregulated by crown rot infection (Hu and Shu, 2021). By analyses of the cis-elements in the promoters of all the five genes, several “defense and stress responsive” and “salicylic acid responsive” elements were identified. The parallel expression patterns in response to diverse stress factors observed for all WHIRLY genes in these three dicot plants suggest that all WHIRLY genes have similar functions during stress in these plants (Hu and Shu, 2021).

For Arabidopsis, it has been postulated that WHIRLIES control genes activated by salicylic acid and containing PB elements in their promoters (Desveaux et al., 2004, 2005). However, these were predictions, and although responses to high light involve salicylic acid signaling (Karpinski et al., 2013), an analysis of global gene expression performed with the *why1tilwhy3* mutant under high light conditions did not show the expected changes in gene expression (Lepage et al., 2013). Moreover, Lepage et al. (2013) reported that plastid genome instability increased when the *why1tilwhy3* mutant was combined with the *polIb-1* mutant, leading to ROS production and retrograde signaling and finally to genetic reprogramming and adaptation to oxidative stress (Lepage et al., 2013). The authors concluded that the genetic reprogramming occurring in the triple mutant is not due to the individual genetic backgrounds of *polIb-1* and *why1tilwhy3*, but instead is induced by the production of ROS resulting from enhanced genome instability in the triple mutant (Supplemental Information S9 in Lepage et al., 2013).

The most striking changes in abundances of transcripts in the barley WHIRLY1 deficient plants (Comadira et al., 2015) have been re-evaluated based on recent barley gene annotations (Supplementary Table 2). In accordance with the delayed chloroplast development of the WHIRLY1 deficient barley plants (Krupinska et al., 2019), the former and the new annotations revealed that the expression of genes encoding the S18 and L23 subunits of the 70S ribosomes is enhanced (Supplementary Table 2). Both subunits are essential for ribosome formation in green plants (Rogalski et al., 2006; Tiller and Bock, 2014). With regard to the dramatic decrease in the level of mRNA encoding the eukaryotic translation factor 4A (Comadira et al., 2015), WHIRLY1 might also promote translation at 80S ribosomes. Increased expression levels of genes encoding components of the chloroplast NADH complex (Supplementary Table 2) might indicate oxidative stress.

Among the genes newly identified to have an enhanced expression level in the barley WHIRLY1 deficient plants by re-evaluation of the barley gene annotations, is the gene encoding RNase J (RNJ) which belongs to the metallo-ß-lactamase family (Supplementary Table 2). RNJ is an abundant protein of nucleoids isolated from maize proplastids (Majeran et al., 2012). This RNase displays endo- and exonuclease activities and is conserved among bacteria, archaea, and chloroplasts. In plants, it has a GT-1 domain that was also found in transcription factors that function in light and stress responses (Halpert et al., 2019). In Arabidopsis, RNJ is required for embryogenesis and chloroplast development (Chen et al., 2015). Disturbance of chloroplast development was proposed to be the reason for the observed abortion of embryos. RNJ promotes chloroplast development by degrading aberrant RNA, such as antisense RNA produced by relaxed transcription in plastids (Sharwood et al., 2011; Hotto et al., 2020).

WHIRLY proteins might control the expression of target genes in cooperation with interacting transcription factors that have partly overlapping binding sequences with WHIRLIES. For some of the WHIRLY target genes, that is, *WRKY53* (Miao et al., 2013), it has been shown that they encode the transcription factors that were shown to interact with a WHIRLY protein, suggesting that WHIRLIES might aid in autoregulation of such genes. Vice versa, concerning the cis-elements identified in promoters of WHIRLY genes, such as W-boxes and GATA elements, it also seems that WHIRLY interacting transcription factors might also control the expression of WHIRLY genes, for example, transcriptional activation of *MeWHIRLY3* by MeWRKY75 (Liu et al., 2018). A complex consisting of MeWHIRLY3 and MeWRKY75 was found to activate genes with PB elements (Liu et al., 2018).

## Stress Resistance of Plants With Altered Abundances of Whirlies

In all species studied so far, high levels of one or the other WHIRLY protein were observed to promote resistance toward various pathogens. To increase stress resistance, different *WHIRLY* genes have been overexpressed in agronomically important plants (Table 2). Tobacco and tomato plants overexpressing *WHIRLY* genes, for example, have a higher resistance toward bacterial and fungal pathogens (Table 2), and overexpression of all three cassava *WHIRLY* genes enhanced resistance against cassava bacterial blight. In contrast, virus-induced gene silencing decreased resistance (Liu et al., 2018). Accordingly, Arabidopsis *TILLING why1* mutants with reduced binding of WHIRLY1 to the PB element (Table 1) showed reduced resistance toward *Peronospora parasitica* (Desveaux et al., 2004), and barley RNAi-WHIRLY1 plants with reduced expression *HvWHIRLY1* have reduced resistance toward powdery mildew (Hensel et al., unpublished). These results are in accordance with the characterization of WHIRLY1 as a transcriptional activator of pathogen response genes as a consequence of salicylic acid signaling (Desveaux et al., 2000, 2004). It has been suggested that WHIRLY1 moves to the nucleus upon oxidative stress-induced production of salicylic acid inside chloroplasts. This pathway would parallel SA-signaling by NPR1, which moves from the cytoplasm to the nucleus in response to salicylic acid and binds to pathogen response gene promoters in the nucleus (Foyer et al., 2014). Among the genes activated in Arabidopsis during pathogen defense is the gene encoding isochorismate synthase 1 (Lin et al., 2020), the key enzyme of the plastid located part of the salicylic acid biosynthesis pathway (Rekhter et al., 2019). Likely, WHIRLY1 and NPR1 have an additive effect on the expression of PR genes as proposed already by Desveaux et al. (2004, 2005), and WHIRLY1 is likely responsible for defense reactions independent of NPR1 (Vlot et al., 2009; An and Mou, 2011; Janda and Ruelland, 2015).

The consistent results on WHIRLY-dependent biotic stress resistance in different species contrasts with the diverse effects of WHIRLY proteins on abiotic stress resistance. While barley RNAi *WHIRLY1* knockdown plants have been reported to have an enhanced resistance toward drought (Janack et al., 2016), cassava plants with silenced *WHIRLY1* (Yan et al., 2020) and tomato lines with silenced *WHIRLY2* showed a reduced drought tolerance (Meng et al., 2020; Yan et al., 2020). The latter findings are in accordance with the enhanced drought resistance of tobacco *WHIRLY2* overexpressing tobacco plants (Zhao et al., 2018). Drought tolerance is primarily mediated by abscisic acid (ABA), which is also involved in responses to different abiotic stresses and biotic stresses (Kumar et al., 2019). Arabidopsis plants overexpressing *AtWHIRLY1* were shown to be hypersensitive to ABA, while a *why1* mutant showed insensitivity toward ABA (Isemer et al., 2012a). When overexpression was performed with a construct lacking the sequence encoding the plastid targeting sequence of AtWHIRLY1, the plants were as insensitive to ABA as the mutant plants (Isemer et al., 2012a), clearly indicating that the location of WHIRLY1 inside chloroplasts is mandatory for the perception of ABA.

In tomato, overexpression of *WHIRLY1* leads to higher chilling tolerance, coinciding with a higher level of soluble sugars and higher photosynthesis likely due to enhanced expression of *RBCS1* (Zhuang et al., 2020b) as well as to enhanced thermotolerance (Zhuang et al., 2020a). Taken together, these results indicate that the WHIRLY proteins have a positive impact on abiotic stress resistance in dicot plants. In contrast, the situation might be more complex in monocot plants, because it is impossible to distinguish direct effects of monocot WHIRLY1 proteins from indirect effects *via* their impacts on organelle DNA compaction (Oetke et al., 2022).

## Putative Mechanisms Underlying ROS Management and Regulation of Stress Resistance by Whirlies

Several studies showed that the changes in abiotic stress resistance of plants with an altered abundance of WHIRLIES are accompanied by changes in organellar ROS levels. Enhanced chloroplast ROS levels were measured by EPR spectroscopy in barley plants with an RNAi-mediated knockdown of *WHIRLY1* grown at high light intensity (Swida-Barteczka et al., 2018). Higher ROS levels were also reported for old leaves of the Arabidopsis *why1* mutant (Lin et al., 2019) and the *why1tilwhy3polIb-1* triple mutant (Lepage et al., 2013). Tomato RNAi*-WHIRLY2* lines were shown to accumulate more ROS during drought and have a lower alternative oxidase activity (Meng et al., 2020). In contrast, transgenic tobacco plants overexpressing *SlWHIRLY2* have reduced ROS levels and enhanced the activities of enzymes of the antioxidative system (SOD, APX; Zhao et al., 2018).

Albeit there is consensus about the suppression of ROS production by WHIRLIES in dicot and monocot species, the suggested sources of ROS and their effectiveness in stress responses in WHIRLY deficient plants were reported to differ. While in barley WHIRLY1 deficient plants, enhanced ROS production is obviously due to an inefficient photosynthetic apparatus (Swida-Barteczka et al., 2018), the enhanced level of ROS in the Arabidopsis *why1til3polIb-1* mutant (Parent et al., 2011; Lepage et al., 2013) was suggested to be a consequence of plastid genome instability coinciding with the appearance of variegated leaves in a portion of the *why1tilwhy3* mutant (Maréchal et al., 2009). Variegation in the mutant was reported to occur more frequently when plants were grown in high light (Guan et al., 2018) and might be caused by inequal resistance of individual plastids to photooxidative stress reported for the *immutans* mutant of Arabidopsis (Yu et al., 2007). It seems that plastids can tolerate a certain level of rearranged DNA molecules as these are also detectable in green sectors of the leaves (Maréchal et al., 2009) while their accumulation gives rise to non-functional plastids. Moreover, ROS production can be promoted by treating the Arabidopsis *why1tilwhy3polIb* mutant plants with ciprofloxacin that induces double-strand break production by inhibition of gyrases during replication (Lepage et al., 2013). The findings suggest that plastid genome instability leads to an increase in the production of ROS. The redox imbalance correlates with altered nuclear gene expression, as various abiotic stress-related genes required for acclimation, that is, *ELIP2*, NUDt4, *ACS6*, *UPOX*, *Armadillo repeat* AT3G06530, were shown to be upregulated. Lepage et al. (2013) postulated that plastid genome instability induces an oxidative burst that favors adaptation to subsequent oxidative stress. This idea got recent support by studies with the triple mutant *recA1why1tilwhy3* and ciprofloxacin. In this mutant, ROS production is promoted by DNA damage in an otherwise non-stress situation. ROS was shown to induce retrograde signaling affecting endoreplication and cell cycle *via* regulation of the nuclear SOG1 transcription factor required for plant growth and development (Duan et al., 2020).

In the *why1tilwhy3* double mutant and the triple mutants prepared by crosses of the double mutant with *polIb* and *recA*, respectively, ROS production obviously induced changes in nuclear gene expression, enabling a light acclimation response. In contrast, in WHIRLY1 deficient plants of barley, ROS production is not able to induce acclimation. Instead, these plants suffer oxidative stress when grown at high irradiance (Swida-Barteczka et al., 2018) and cannot accelerate senescence in response to light (Kucharewicz et al., 2017). Leaves of the plants indeed lack typical reactions associated with acclimation to high light (Saeid Nia et al., 2022). The plants are not only insensitive to light, but also to the supply of water (Janack et al., 2016) and nitrogen (Comadira et al., 2015). In WHIRLY1 deficient barley plants grown under nitrogen-deficient conditions, leaf chlorophyll content and photosynthesis were higher than in the wild type (Comadira et al., 2015). The results suggest that the barley plants are compromised in the measurement of light intensity and cannot activate the required stress defense programs. The function of WHIRLY1 in sensing environment is in accordance with the reduced stress resistance reported for WHIRLY1 deficient dicot plants (Table 2).

The capability of the Arabidopsis *why1tilwhy3* mutant to activate stress responses (Lepage et al., 2013) might be due to the presence of the truncated AtWHIRLY3 including the KGKAAL motif (see above and Supplementary Figure 2). In comparison, the reduced resistance to DNA damage as reported for the *why1tilwhy3* mutant might be at least partly caused by the lack of motifs downstream of the DNA-binding motif (Figure 1).

Considering that WHIRLY proteins are also present in the nucleus where they might aid in the regulation of the expression stress-related genes, it is difficult to distinguish the impact of organellar ROS production and subsequent retrograde signaling on nuclear gene regulation from the direct effect of chloroplast-to-nucleus translocation of WHIRLY proteins on stress resistance.

## Concluding Remarks and Outlook

WHIRLIES apparently have a high impact on plant development and stress resistance. By binding to ssDNA and RNA, WHIRLY proteins influence diverse DNA and RNA associated processes in the organelles and connect them with the responsiveness to environment being important for both development and stress resistance. During stress, WHIRLIES participate in retrograde signaling in two ways: (1) retrograde signaling initiated by perturbation of electron transport leading to ROS production and hormone signaling and (2) directly as a chloroplast-to-nucleus translocating protein (Bobik and Burch-Smith, 2015). This latter pathway is likely activated by salicylic acid or other stress-related signal compounds, such as ROS and methylerythritol cyclopyrophosphate (MEcPP; Xiao et al., 2012, 2013). In bacteria, MEcPP produced during oxidative stress was reported to release histone-like proteins from DNA (Artsatbanov et al., 2012). In plants, MEcPP could be the link between the generation of chloroplast stress signals and the translocation of WHIRLY1 from chloroplasts to the nucleus (Foyer et al., 2014).

Putative and confirmed WHIRLY interacting proteins involve on one hand organellar proteins serving as components of the photosynthetic machinery, nucleoids, ribosomes, and enzymes of the primary metabolism, and on the other hand transcription factors in the nucleus. These numerous interactions might accommodate the coordination of plant functions at different levels during the plants’ response to environment.

WHIRLY proteins of different species possess different motifs besides the highly conserved WHIRLY domain responsible for binding ssDNA and oligomerization. The sequence diversity outside of the WHIRLY domain indicates that WHIRLIES can serve various purposes in different species. Likely, decisions among the multiple functions of WHIRLIES and their subcellular distribution involves various posttranslational modifications which remain to be determined in the different species.

The growing knowledge on the impact of WHIRLY proteins on numerous processes affecting development and resistance to diverse abiotic and biotic stresses indicate that *WHIRLIES* belong to the group of “multi-role pleiotropic genes” which are expected to be valuable targets for developing crop plants with an inbuilt tolerance to multiple stresses (Husaini, 2022). It stands to reason that WHIRLIES’ impact on resistance to multiple stresses depends on their dynamic localization in organelle nucleoids and the nucleus.

As major nucleoid-associated proteins, WHIRLIES might operate in “epigenetic reprogramming” of organelle genomes. In humans/mammals, reversible NAP-mediated changes in the structure and function of mitochondrial nucleoids were observed to affect the mitochondria–nucleus cross-talk, being of pivotal importance for an efficient operation of mitochondria in energy-demanding tissues (Arunachalam et al., 2021). Mitochondrial epigenomics and its impact on mitochondria–nucleus cross-talk offered novel perspectives for studying diseases, such as cancer (Sharma et al., 2021). A deep understanding of the mechanisms underlying the impact of WHIRLIES on nucleoid structure and the consequences for photosynthesis and respiration will increase the chances to develop plants with higher productivity and stress resistance.

## Author Contributions

KK has outlined and mainly written the manuscript. CD performed microscopy research and prepared the figures. SF performed sequence analyses and evaluated published data sets. GH re-evaluated published data on gene expression. All authors contributed to the article and approved the submitted version.

## Funding

The joint research of GH and KK on WHIRLY proteins is funded by the German Research Foundation (HE6432/4-1, KR1350/25-1). GH is supported by funding of the Deutsche Forschungsgemeinschaft (DFG, German Research Foundation) under Germany’s Excellence Strategy—EXC-2048/1—project ID 390686111.

## Conflict of Interest

The authors declare that the research was conducted in the absence of any commercial or financial relationships that could be construed as a potential conflict of interest.

## Publisher’s Note

All claims expressed in this article are solely those of the authors and do not necessarily represent those of their affiliated organizations, or those of the publisher, the editors and the reviewers. Any product that may be evaluated in this article, or claim that may be made by its manufacturer, is not guaranteed or endorsed by the publisher.
