# Supplementary material for: WHIRLIES Are Multifunctional DNA-Binding Proteins With Impact on Plant Development and Stress Resistance
Source: Front Plant Sci. 2022 Apr 21;13:880423. doi: 10.3389/fpls.2022.880423 (PMC9070903; doi:10.3389/fpls.2022.880423)
Supplement: Supplementary file 1 [file Data_Sheet_1.pdf]

SUPPLEMENTARY MATERIAL

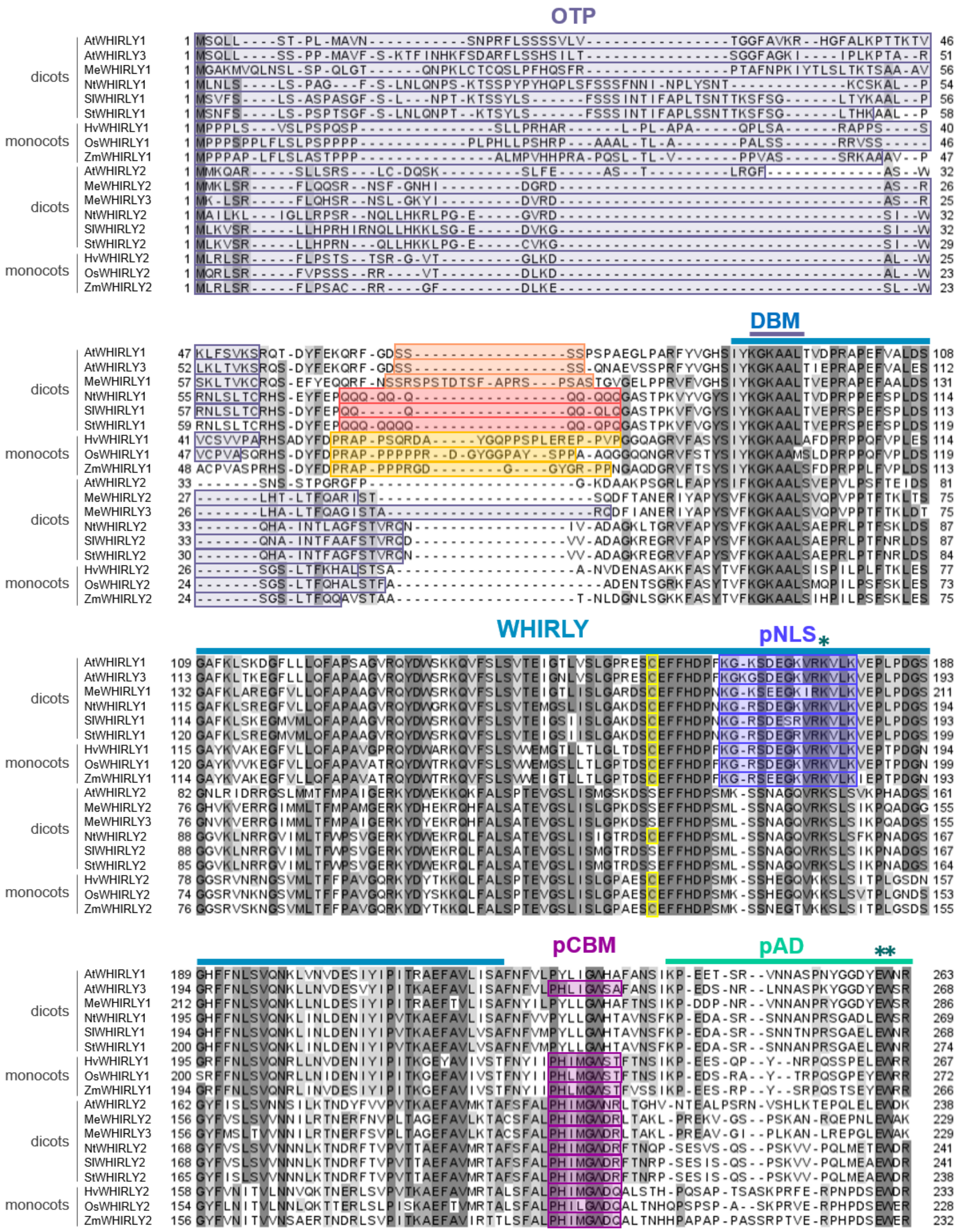

SUPPLEMENTARY FIGURE 1 Alignment of amino acid sequences of selected WHIRLY proteins.

AtWHIRLY1 (*Arabidopsis thaliana*, NP_172893.1), AtWHIRLY2 (*Arabidopsis thaliana*, NP_177282.2), AtWHIRLY3 (*Arabidopsis thaliana*, NP_178377), MeWHIRLY1 (*Manihot esculenta*, XP_021603431.1), MeWHIRLY2 (*Manihot esculenta*, XP_021600346.1), MeWHIRLY3 (*Manihot esculenta*, XP_021599713.1), NtWHIRLY1 (*Nicotiana tabacum,* XP_016453689.1), NtWHIRLY2 (*Nicotiana tabacum,* XP_016511175.1), SlWHIRLY1 (*Solanum lycopersicum*, AFY24240.1), SlWHIRLY2 (*Solanum lycopersicum*, XP_010313085.1), StWHIRLY1 (*Solanum tuberosum,* NP_001275155.1), StWHIRLY2 (*Solanum tuberosum,* NP_001275393.1), HvWHIRLY1 (*Hordeum vulgare*, BAJ96655), HvWHIRLY2 (*Hordeum vulgare*, XP_044950776.1), OsWHIRLY1 (*Oryza sativa*, BAD68418.1), OsWHIRLY2 (*Oryza sativa*, NP_001045956.1), ZmWHIRLY1 (*Zea mays,* NP_001123589.1), ZmWHY2 (*Zea mays,* NP_001152589.2).

The Alignment was done with TcoffeeWS in Jalview software. The Percentage of identity is shown in grey. The WHIRLY domain is marked with a blue line. The organelle targeting peptides (OTP) were predicted with TargetP-2.0 (https://services.healthtech.dtu.dk/-service.php?TargetP-2.0) or UniProt, (https://www.uniprot.org/), the putative nuclear localization signal (pNLS) with NLStradamus (http://www.moseslab.csb.utoronto.ca/NLStradamus/) and the putative copper binding motif (pCBM) with Motif Scan (https://myhits.sib.swiss/cgi-bin/motif_scan). The putative auto-regulatory domain (pAD) was defined by Desveaux et al., 2005. Asterisks mark highly conserved amino acid residues corresponding to the interacting Lys188, Glu271 and Trp272 of StWHIRLY1 (Desveaux et al. 2005). The cysteine residue in the WHIRLY domain is coloured in yellow; the proline-, serine- or glutamine-rich sequences are orange. DBM – DNA binding domain.

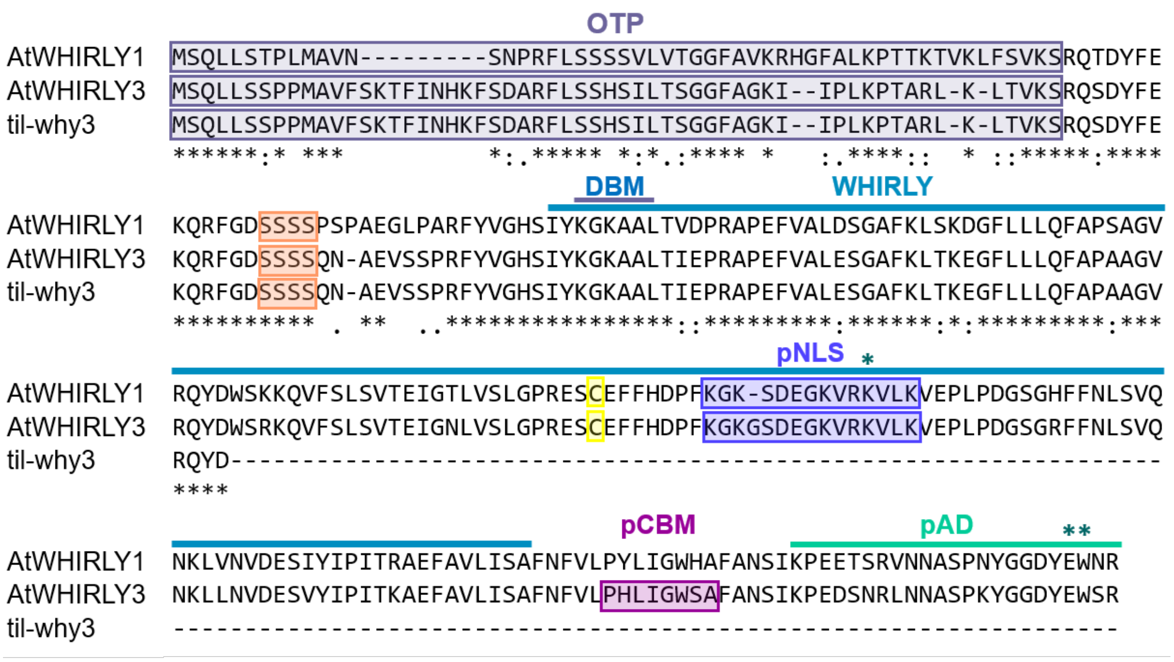

SUPPLEMENTARY FIGURE 2 Alignment of the truncated WHIRLY3 of the *tilwhy3* mutant with the amino acid sequences of AtWHIRLY1 (NP_172893.1) and AtWHIRLY3 (NP_178377).

The amino acid sequence of the truncated WHIRLY3 in the *tilwhy3* mutant from the Arabidopsis TILLING project (ATP) (Till et al., 2003;Maréchal et al., 2009) still contains the DNA binding motif KGKAAL.

SUPPLEMENTARY Table 1: Experimentally identified interaction partners of WHIRLIES.
CP - chloroplasts, Cyt - cytosol, cytRib - cytosolic ribosomes, Mito - mitochondria, N - nucleus, PM - plasma membrane, V - vacuole. Methods: BiFC - bimolecular fluorescence complementation, CoIP - coimmunoprecipitation, CrY2H-seq - Cre reporter-mediated yeast two-hybrid coupled with next-generation sequencing, Y2H - yeast two-hybrid assay.

WHIRLY	Species	Interacting protein	full name and synonyms	TAIR/ GeneBank Number	UniProt Number	Functions	Locali-zation	Method	References
**AtWHY1** (AT1G14410)	*Arabidopsis thaliana*	CIPK14	CBL-interacting protein kinase 14, PKS24, Serine/threonine protein kinase 1, SNF1-related protein kinase 3.15 (SNRK3.15), SOS2-like protein kinase 24, SR1	AT5G01820	Q9LZW4	CIPK serine-threonine protein kinases interact with CBL proteins. Binding of a CBL protein to the regulatory NAF domain of CIPK protein lead to the activation of the kinase in a calcium-dependent manner.	Cyt, N	Y2H	(Ren et al., 2017)
		LHCA1	Light harvesting complex of photosystem I 1, Chlorophyll a-b binding protein 6	AT3G54890	Q01667	Component of the light harvesting complex associated with photosystem I.	CP	Y2H, CoIP, BiFC	(Huang et al., 2017)
		TERT	Telomerase reverse transcriptase	AT5G16850	Q9SPU7	Catalytic subunit of telomerase reverse transcriptase, involved in telomere homeostasis.	N	Tandem affinity purifi-cation	(Majerska et al., 2017)
		WRKY53		AT4G23810	Q9SUP6	Member of WRKY Transcription Factor, Group III. Interacts specifically with the W box. Involved in regulation of early events of leaf senescence, expression of ESR/ESP, resistance to P.syringae and of jasmonic acid-induced expression of PDF1.2.	N	CrY2H-seq	(Trigg et al., 2017a)
**AtWHY1** (AT1G14410) / **AtWHY3** (AT2G02740)	*Arabidopsis thaliana*	2-Cys PrxA	2-Cys Peroxiredoxin A, 2CPA, BAS1	AT3G11630	Q96291	Functions in redox cascade with TrxL2 via the FTR/Trx pathway to mediate the light-responsive reductive control of target proteins. Plays a role in cell protection against oxidative stress by detoxifying peroxides.	CP	pull down	(Liebthal et al., 2020)
		RNase H1C	Ribonuclease H1C, RNH1C	AT1G24090	F4I7R5	Maintenance of plastid genome integrity	CP	Co-IP	(Yang et al., 2017)
**AtWHY2** (AT1G71260)	*Arabidopsis thaliana*	AGL74	AGAMOUS-LIKE 74, F21D18.12	AT1G48150	Q9LNG8	MADS-box transcription factor family protein, RNA polymerase II-specific	N	CrY2H-seq	(Trigg et al., 2017b)
		AGL84	AGAMOUS-LIKE 84	AT5G49420	Q7X9H5	MADS-box transcription factor family protein, RNA polymerase II-specific	N	CrY2H-seq	(Trigg et al., 2017b)
		ATARCA	Receptor for activated C kinase 1A (RACK1A), Suppressor of acaulis 53 (SAC53)	AT1G18080	O24456	Major component of the RACK1 regulatory proteins that play a role in multiple signal transduction pathways. Involved in multiple hormone responses and developmental processes.	CP, N, Cyt, cytRib, PM	Y2H	(Guo et al. 2019)
		ODB1	RAD52-1	AT1G71310	Q9FVV7	Plant-specific single-stranded DNA-binding protein required for efficient heterologous recombination-dependent DNA repair in nuclear and mitochondrial compartments. Forms large nucleo-protein complexes with WHY2 in mitochondria.<br>Involved in the hydrolytic splicing pathway in mitochondrion.	Mito, N	DNA-affinity purify-cation	(Janicka et al., 2012)

**AtWHY2** (AT1G71260)	*Arabidopsis thaliana*	SEX1	Starch excess 1, Alpha-glucan water dikinase 1, GWD1, SOP1	AT1G10760	Q9SAC6	Phytohormone signal integration. Required for starch degradation. Mediates the incorporation of phosphate into starch-like alpha-glucan. Involved in cold-induced freezing tolerance.	CP	Y2H	(Altmann et al., 2020)
		SYP23	Syntaxin of plants 23	AT4G17730	A8MS65	SNAP receptor activity, involved in vesicle-mediated transport	PM, N, Cyt, V	Y2H	(Klopffleisch et al., 2011)
		TGA1	TGACG sequence-specific binding protein 1	AT5G65210	Q39237	Transcription factor, redox-controlled regulator of systemic acquired resistance	N	CrY2H-seq	(Trigg et al., 2017b)
**AtWHY2** (AT1G71260) / **AtWHY3** (AT2G02740)	*Arabidopsis thaliana*	ARF19	Auxin response factor 19 ARF11, Indole-3-acetic acid inducible 22 (IAA22)	AT1G19220	Q8RYC8	Transcriptional factor that bind specifically to auxin-responsive promoter elements (AuxREs). Could act as transcriptional activator or repressor. Involved in ethylene responses.	N	CrY2H-seq	(Trigg et al., 2017b)
**AtWHY3** (AT2G02740)	*Arabidopsis thaliana*	60S ribosomal protein L12-2		AT3G53430	Q9LFH5	Ribosomal protein L11 family protein. Binds directly to 26S ribosomal RNA.	Cyt, cytRib	Y2H	(Isemer, 2013)
		ACO1	Aconitase 1, Aconitate hydratase 1	AT4G35830	Q42560	Catalyzes the isomerization of citrate to isocitrate via cis-aconitate. This contributes to oxidative stress tolerance.	Mito, Cyt	Y2H	(Isemer, 2013)
		BCCP1	BIOTIN CARBOXYL-CARRIER PROTEIN 1, CHLOROPLASTIC ACETYLCOENZYME A CARBOXYLASE 1 (CAC1A)	AT5G16390	Q42533	biotin carboxyl-carrier subunit of the multi-enzyme plastidial acetyl-coenzyme A carboxylase complex, involved in fatty acid biosynthesis	CP	CoIP	(Isemer, 2013)
		CLPC1	Chaperone of Clp-protease, HSP93-V	AT5G50920	Q9FI56	Molecular chaperone that hydrolyzes ATP and is associated with the chloroplast protein import apparatus. May interact with a ClpP-like protease involved in degradation of denatured proteins in the chloroplast. Involved in the regulation of chlorophyll b biosynthesis.	CP	CoIP	(Isemer, 2013)
		CysC1	Bifunctional L-3-cyanoalanine synthase/cysteine synthase C1, ß-Cyanoalanine synthase C1 (CAS-C1)	AT3G61440	Q9S757	The cysteine synthase isomer CysC1 is less effective in cysteine biosynthesis.	Mito	Y2H	(Isemer, 2013)
		Cytochrome f	Photosynthetic electron transfer A, PetA	ATCG00540	P56771	Component of the cytochrome b6-f complex, which mediates electron transfer between photosystem II (PSII) and photosystem I (PSI), cyclic electron flow around PSI, and state transitions.	CP	CoIP	(Isemer, 2013)
		FIB1a	Fibrillin 1A, Plastoglobulin 35 (PGL35), Plastid-lipid-associated protein 1	AT4G04020	O81439	Involved in abscisic acid-mediated photoprotection. Localisation in plastoglobulins and thylakoid membranes	CP	CoIP	(Isemer, 2013)
		FIB4	fibrillin			Localisation in plastoglobules and thylakoid membranes	CP	CoIP	(Isemer, 2013)
		GATA14	GATA transcription factor 14	AT3G45170	Q9M1U2	Member of the GATA factor family of zinc finger transcription factors. May be involved in the regulation of some light-responsive genes	N	CrY2H-seq	(Trigg et al., 2017b)
		LHCB5	Light harvesting complex of photosystem II 5, Chlorophyll a-b binding protein CP26	AT4G10340	Q9XF89	Light-harvesting chlorophyll a/b binding protein of the antenna system of the photosynthetic apparatus.	CP	CoIP	(Isemer, 2013)

**AtWHY3** (AT2G02740)	*Arabidopsis thaliana*	PPR4	Pentatricopeptide repeat 4	AT5G04810	Q0WMY5	Essential during the early stages of embryo development. Acts in the plastid nucleoids as the factor responsible of rps12 intron 1 trans-splicing and, indirectly, in the assembly of 70S ribosomes and plastid translation.	CP (TAC)	CoIP	(Isemer, 2013)
		PRPS3	Plastid ribosomal protein of the 30S subunit 3, 30S ribosomal protein S3	ATCG00800	P56798	Chloroplast ribosomal protein, constituent of the small subunit of the ribosomal complex.	CP (TAC)	CoIP	(Isemer, 2013)
		PRPS5	Plastid ribosomal protein of the 30S subunit 5, Embryo defective 3113 (EMB3113), 30S ribosomal protein S5, SCABRA 1 (SCA1)	AT2G33800	P93014	Plastid-type ribosomal protein that functions as a structural component of the 70S plastid ribosome. Binds directly to 16S ribosomal RNA.	CP (TAC)	CoIP	(Isemer, 2013)
		PTAC4	Plastid transcriptionally active 4, Vesicle-inducing protein in plastids 1 (VIPP1), IM30	AT1G65260	O80796	Required for thylakoid membrane biogenesis and plastid vesicle formation.	CP (TAC)	CoIP	(Isemer, 2013)
		WHY1	WHIRLY 1	AT1G14410	Q9M9S3	see this review	CP, N	CrY2H-seq	(Trigg et al., 2017b)
		WOX13	WUSCHEL-related homeobox 13, HB-4	AT4G35550	O81788	Transcription factor which may be involved in developmental processes.	N	CrY2H-seq	(Isemer, 2013;Trigg et al., 2017b)
**MeWHIRLY1 / MeWHIRLY2 / MeWHIRLY3**	*Manihot esculenta*	WRKY75	WRKY transcription factor 75	KT827650	A0A140-H8S9	DNA-binding transcription factor	N	Y2H + BiFC	(Liu et al., 2018)
**SlWHIRLY2**	*Solanum lycopersicum*	RECA2	Recombinase A homolog 2	NP_001307305	B1N670	Important for mtDNA recombination and repair	Mito	luciferase comple-mentation assay CoIP	(Meng et al., 2020)
**ZmWHIRLY1**	*Zea mays*	CRS1		AF290414.1	Q9FYT6	Chloroplastic group IIA intron splicing facilitator. Required for the splicing of group IIA introns in chloroplasts, and especially for atpF, by regulating the intron folding. Forms splicing particles with RNA. Also involved in chloroplast protein translation.	CP	CoIP	(Prikryl et al., 2008)

References

Altmann, M., Altmann, S., Rodriguez, P.A., Weller, B., Vergara, L.E., Palme, J., De La Rosa, N.M., Sauer, M., Wenig, M., Villaecija-Aguilar, J.A., Sales, J., Lin, C.W., Pandiarajan, R., Young, V., Strobel, A., Gross, L., Carbonnel, S., Kugler, K.G., Garcia-Molina, A., Bassel, G.W., Falter, C., Mayer, K.F.X., Gutjahr, C., Vlot, A.C., Grill, E., and Falter-Braun, P. (2020). Extensive signal integration by the phytohormone protein network. *Nature* 583**,** 271-276.

Huang, D.M., Lin, W.F., Deng, B., Ren, Y.J., and Miao, Y. (2017). Dual-Located WHIRLY1 Interacting with LHCA1 Alters Photochemical Activities of Photosystem I and Is Involved in Light Adaptation in Arabidopsis. *International Journal of Molecular Sciences* 18.

Isemer, R. (2013). *Die WHIRLY-Proteine von Arabidopsis thaliana.* Mathematical and Natural Sciences Faculty, *CAU* Kiel.

Janicka, S., Kuhn, K., Le Ret, M., Bonnard, G., Imbault, P., Augustyniak, H., and Gualberto, J.M. (2012). A RAD52-like single-stranded DNA binding protein affects mitochondrial DNA repair by recombination. *Plant Journal* 72**,** 423-435.

Klopffleisch, K., Phan, N., Augustin, K., Bayne, R.S., Booker, K.S., Botella, J.R., Carpita, N.C., Carr, T., Chen, J.G., Cooke, T.R., Frick-Cheng, A., Friedman, E.J., Fulk, B., Hahn, M.G., Jiang, K., Jorda, L., Kruppe, L., Liu, C.G., Lorek, J., Mccann, M.C., Molina, A., Moriyama, E.N., Mukhtar, M.S., Mudgil, Y., Pattathil, S., Schwarz, J., Seta, S., Tan, M., Temp, U., Trusov, Y., Urano, D., Welter, B., Yang, J., Panstruga, R., Uhrig, J.F., and Jones, A.M. (2011). Arabidopsis G-protein interactome reveals connections to cell wall carbohydrates and morphogenesis. *Molecular Systems Biology* 7.

Liebthal, M., Schuetze, J., Dreyer, A., Mock, H.P., and Dietz, K.J. (2020). Redox conformation-specific protein-protein interactions of the 2-cysteine peroxiredoxin in Arabidopsis. *Antioxidants* 9, 515.

Liu, W., Yan, Y., Zeng, H.Q., Li, X.L., Wei, Y.X., Liu, G.Y., He, C.Z., and Shi, H.T. (2018). Functional characterization of WHY-WRKY75 transcriptional module in plant response to cassava bacterial blight. *Tree Physiology* 38**,** 1502-1512.

Majerska, J., Schrumpfova, P.P., Dokladal, L., Schorova, S., Stejskal, K., Oboril, M., Honys, D., Kozakova, L., Polanska, P.S., and Sykorova, E. (2017). Tandem affinity purification of AtTERT reveals putative interaction partners of plant telomerase in vivo. *Protoplasma* 254**,** 1547-1562.

Maréchal, A., Parent, J.S., Veronneau-Lafortune, F., Joyeux, A., Lang, B.F., and Brisson, N. (2009). Whirly proteins maintain plastid genome stability in Arabidopsis. *Proceedings of the National Academy of Sciences of the United States of America* 106**,** 14693-14698.

Meng, C., Yang, M.M., Wang, Y.X., Chen, C., Sui, N., Meng, Q.W., Zhuang, K.Y., and Lv, W. (2020). SlWHY2 interacts with SlRECA2 to maintain mitochondrial function under drought stress in tomato. *Plant Science* 301**,** 8.

Prikryl, J., Watkins, K.P., Friso, G., Van Wijk, K.J., and Barkan, A. (2008). A member of the Whirly family is a multifunctional RNA- and DNA-binding protein that is essential for chloroplast biogenesis. *Nucleic Acids Research* 36**,** 5152-5165.

Ren, Y.J., Li, Y.Y., Jiang, Y.Q., Wu, B.H., and Miao, Y. (2017). Phosphorylation of WHIRLY1 by CIPK14 Shifts Its Localization and Dual Functions in Arabidopsis. *Molecular Plant* 10**,** 749-763.

Till, B.J., Reynolds, S.H., Greene, E.A., Codomo, C.A., Enns, L.C., Johnson, J.E., Burtner, C., Odden, A.R., Young, K., Taylor, N.E., Henikoff, J.G., Comai, L., and Henikoff, S. (2003). Large-scale discovery of induced point mutations with high-throughput TILLING. *Genome Research* 13**,** 524-530.

Trigg, S.A., Garza, R.M., Macwilliams, A., Nery, J.R., Bartlett, A., Castanon, R., Goubil, A., Feeney, J., O'malley, R., Huang, S.S.C., Zhang, Z.Z., Galli, M., and Ecker, J.R. (2017b). CrY2H-seq: a massively multiplexed assay for deep-coverage interactome mapping. *Nature Methods* 14**,** 819-825.

Yang, Z., Hou, Q.C., Cheng, L.L., Xu, W., Hong, Y.T., Li, S., and Sun, Q.W. (2017). RNase H1 Cooperates with DNA Gyrases to Restrict R-Loops and Maintain Genome Integrity in Arabidopsis Chloroplasts. *Plant Cell* 29**,** 2478-2497.

SUPPLEMENTARY TABLE 2 Genes with altered expression in RNAi-mediated WHIRLY1 knockdown plants of barley

Data published by Comadira et al. (2015, *Plant Physiology* 168, 1140-1151) have been re-evaluated based on recent barley gene annotations (https://doi.org/10.1093/plcell/koab077). Only the genes identified with high confidence (Supplemental Table S1 in Comadira et al. 2015) are listed.

Accession	New ID	Transcript abundance	Protein	Functional context
MLOC_26926.1	HORVU.MOREX.r3.6HG0555150.1	+4.2	unknown	
MLOC_5848.1	HORVU1Hr1G048160	+2.4	50S plastid ribosomal protein L32	plastid translation
AK376535	HORVU.MOREX.r3.6HG0555260.1	-5.2	F-box containing protein	protein degradation by the ubiquitin proteasome system (UPS),
MLOC_53680.1	HORVU3Hr1G049980	+3.5	Histone acetylase (HAT), hobo, Ac, Ram3 transposable elements	chromatin remodeling, epigenetics
AK3662522	HORVU.MOREX.r3.6HG0629240.1	+2.6	O-methyltransferase	lignin biosynthesis, defense
MLOC_44822.1	HORVU.MOREX.r3.1HG0055560.1	+2.3	RNaseJ, a metallo-beta-lactamase family protein	degradation of aberrant RNA, chloroplast development
MLOC_68719.1	HORVU.MOREX.r3.3HG0293790.1	+2.3	eukaryotic translation initiation factor 3 subunit 6-interacting protein	cytosolic translation
MLOC_514.1	HORVU.MOREX.r3.UnG0816080.1	+2.1	30S plastid ribosomal protein S18	plastid translation
MLOC_32552.1	HORVU3Hr1G074860	+1.6	50S plastid ribosomal protein L23	Plastid translation
MLOC_37150.2	HORVU.MOREX.r3.5HG0445760.1	-2.3	F-box protein	protein degradation va the ubiquitin proteasome system (UPS)
MLOC_61005.5	HORVU.MOREX.r3.7HG0702610.1	-3.0	eukaryotic initiation factor 4A (ATP dependent RNA helicase elf4A)	cytosolic translation
MLOC_24854.1	HORVU.MOREX.r3.UnG0815660.1	+2.9	plastid NAD(P)-quinone oxidoreductase chain 4 (NDH4=NdhD, pt encoded)	redox regulation, oxidative stress
MLOC_24776.1	HORVU.MOREX.r3.UnG0815650.1	+2.0	cytochrome c biogenesis protein CcsA2	heme transport
MLOC_25280.1	HORVU.MOREX.r3.UnG0815640.1	+1.7	plastid NAD(P)H-quinone oxidoreductase subunit 5 (NDH5-NdhE, pt-encoded)	redox regulation, oxidative stress
AK251585.1	HORVU.MOREX.r3.3HG0319200.1	+1.6	B3 domain-containing protein	
AK365452	HORVU.MOREX.r3.7HG0650090.1	-4.4	WHIRLY1	
MLOC_7242.2	HORVU.MOREX.r3.1HG0000220.1	-4.0	Lr21, Rp1-like protein	leaf rust resistance
AK357471	HORVU.MOREX.r3.5HG0510530.1	+3.5	major facilitator superfamily protein	
AK248474.1	HORVU.MOREX.r3.7HG0690270.1	+3.0	Cu-Zn superoxide dismutase, chloroplast	redox
MLOC_70674.1	HORVU7Hr1G115130	+1.7	unknown protein	
MLOC_39273.1	HORVU.MOREX.r3.4HG0409180.1	-1.7	glutathione-S-transferase	redox
MLOC_1704.1	HORVU.MOREX.r3.UnG0816560.1	+2.5	DNA-directed RNA polymerase subunit beta (RpoB)	plastid transcription (PEP)
MLOC_24746.1	HORVU.MOREX.r3.UnG0816560.1	+2.3	DNA-directed RNA polymerase subunit beta (RpoB)	plastid transcription (PEP
AK359751	HORVU.MOREX.r3.5HG0477720.1	+3.1	phytochromobilin:ferredoxin oxidoreductase, GUN3	phytochrome biosynthesis
MLOC_16007.1	HORVU.MOREX.r3.2HG0208280.1	+1.8	flavonol 4-sulfotransferase	sulfation of flavonoids
